# Supplementary material for: The Relationship between the IC50 Values and the Apparent Inhibition Constant in the Study of Inhibitors of Tyrosinase Diphenolase Activity Helps Confirm the Mechanism of Inhibition
Source: Molecules. 2022 May 13;27(10):3141. doi: 10.3390/molecules27103141 (PMC9142954; doi:10.3390/molecules27103141)
Supplement: Supplementary file 1 [file molecules-27-03141-s001.zip › molecules-1697898-supplementary.pdf]

The Relationship Between the IC_{50} Values and the Apparent Inhibition Constant in the Study of Inhibitors of Tyrosinase Diphenolase Activity Helps Confirm the Mechanism of Inhibition

Pablo Garcia Molina[1], Francisco Garcia Molina[2*], Jose Antonio Teruel Puche[3], Jose Neptuno Rodriguez Lopez[1], Francisco Garcia Canovas[1], Jose Luis Muñoz Muñoz[4*]

[1] GENZ-Group of Research on Enzymology, Department of Biochemistry and Molecular Biology-A, Regional Campus of International Excellence "Campus Mare Nostrum", University of Murcia, Espinardo, Murcia, Spain.

[2] Department of Anatomía Patológica. Hospital General Universitario Reina Sofía, Av. Intendente Jorge Palacios, 1, 30003 Murcia, Spain.

[3] Department of Biochemistry and Molecular Biology-A, Regional Campus of International Excellence "Campus Mare Nostrum", University of Murcia, Espinardo, Murcia, Spain.

[4] Microbial Enzymology Lab, Department of Applied Sciences, Ellison Building A, University of Northumbria, Newcastle Upon Tyne, UK.

* Correspondence: pacogm@um.es, jose.munoz@northumbria.ac.uk

Table of contents:

Content of materials.	Page
S1. Kinetic analysis.	S-6
S1.1. Catalysis.	S-6
S1.1.1. Tyrosinase catalysis.	S-6
S1.2. Inhibition.	S-6
S1.2.1. Tyrosinase inhibition.	S-7
S1.2.2. Rate equation of tyrosinase inhibition according to Scheme 2.	S-7
S1.2.3. Equations of the initial rate of product formation in the presence of an inhibitor: competitive, non-competitive, uncompetitive and mixed.	S-8
Equation of the degree of inhibition ($\boldsymbol{i}_{\mathbf{D}}^{\mathbf{C}}$) and of $\mathbf{IC}_{\mathbf{50}}^{\mathbf{C}}$ in the presence of a competitive inhibitor.	S-8
Equation of the degree of inhibition ($\boldsymbol{i}_{\mathbf{D}}^{\mathbf{NC}}$) and of $\mathbf{IC}_{\mathbf{50}}^{\mathbf{NC}}$ in the presence of a non-competitive inhibitor.	S-8
Equation of the degree of inhibition ($\boldsymbol{i}_{\mathbf{D}}^{\mathbf{U}}$) and of $\mathbf{IC}_{\mathbf{50}}^{\mathbf{U}}$ in the presence of an uncompetitive inhibitor.	S-9
Equation of the degree of inhibition ($\boldsymbol{i}_{\mathbf{D}}^{\mathbf{M}}$) y de $\mathbf{IC}_{\mathbf{50}}^{\mathbf{M}}$ in the presence of a mixed inhibitor.	S-9
S1.3. Differential equations corresponding to the different inhibition mechanisms.	S-11
S1.3.1. Competitive inhibition.	S-11
S1.3.2. Non-competitive inhibition.	S-11
S1.3.3. Uncompetitive inhibition.	S-12
S1.3.4. Mixed inhibition.	S-13
S1.4. Schemes.	S-14

S1.4.1. Scheme S1. Mechanism of action of tyrosinase on L-Dopa in the presence of an inhibitor that is an alternative substrate.	S-14
S1.4.2. Scheme S2. Mechanism of action of tyrosinase on L-Dopa in the presence of an inhibitor that is a suicide substrate.	S-15
S2. Chemical structures of the inhibitors described in the different figures. Docking of some of these molecules to oxy-tyrosinase or met-tyrosinase.	S-16
S2.1. Figure S1. Derivados de benzimidazothiazolone.	S-16
Figure S2. Docking of (Z)-2-(4-Hydroxybenzylidene)benzo[4,5]imidazo[2,1-*b*]thiazol-3(2*H*)-one to oxy-tyrosinase.	S-17
Figure S3. Docking of (Z)-2-(3,4-Dihydroxybenzylidene)benzo[4,5]imidazo[2,1-*b*]thiazol-3(2*H*)-one to oxy-tyrosinase (A) and met-tyrosinase (B).	S-18
Figure S4. Docking of (Z)-2-(2,4-Dihydroxybenzylidene)benzo[4,5]imidazo[2,1-*b*]thiazol-3(2*H*)-one to oxy-tyrosinase.	S-19
S2.2. Figure S5. Chemical structures of urolithin derivatives.	S-20
Figure S6. Docking of 1,3-Dihydroxy-6*H*-benzo[c]chromen-6-one to *oxy*-tyrosinase.	S-21
Figure S7. Docking of 1,3-Dihydroxy-8-methoxy-6*H*-benzo[c]chromen-6-one to *oxy*-tyrosinase.	S-22
Figure S8. Docking of 2’-(Hydroxymethyl)-[1,1’-biphenyl]-2,4-diol to *oxy*-tyrosinase.	S-23
S2.3. Figure S9. Chemical structure of a cinnamic acid derivative: Trans-3,4-diflurocinnamic acid.	S-24

S2.4. Figure S10. Chemical structure of some indazoles: 7-bromo-1*H*-indazole (a); 6-bromo-1*H*-indazole (b); 4-chloro-1*H*-indazole (c); 6-fluoro-1*H*-indazole (d); 7-fluoro-1*H*-indazole (e).	S-25
S2.5. Figure S11. Chemical structure of benzoic acid derivatives: 2-amino benzoic acid (a); 4-amino benzoic acid (b).	S-26
S2.6. Figure S12. Chemical structure of luteolin.	S-27
Figure S13. Docking of luteolin to oxy-tyrosinase.	S-28
Figure S14. Docking of luteolin to met-tyrosinase.	S-29
S2.7. Figure S15. Chemical structure of propyl gallate.	S-30
S2.8. Figure S16. Chemical structure of polyphenols: sanggenone C (a); oxyresveratrol (b); L-epicatechin (c) and catechin (d).	S-31
Figure S17. Docking of sanggenone C to oxy-tyrosinase.	S-32
Figure S18. Docking of oxyresveratrol to oxy-tyrosinase.	S-33
Figure S19A,B. Docking of L-epicatechin to oxy-tyrosinase (A) and met-tyrosinase (B).	S-34
Figure S20A,B. Docking of catechin to oxy-tyrosinase (A) and met-tyrosinase (B).	S-35
S2.9. Figure S21. Chemical structure of phenetyl cinnamamide derivatives: *N-trans*-caffeoyltyramine (a); *N-trans*-feruloyltyramine (b) and *N-trans*-coumaroyltyramine (c).	S-36
Figure S22A,B. Docking of *N-trans*-caffeoyltyramine to oxy-tyrosinase (A) and met-tyrosinase (B).	S-37
Figure S23. Docking of *N-trans*-feruloyltyramine to oxy-tyrosinase.	S-38
Figure S24. Docking of *N-trans*-coumaroyltyramine to oxy-tyrosinase.	S-39
S2.10. Figure S25. Chemical structure of cinnamc acid ester derivatives: (*E*)-2-acetyl-5-methoxyphenyl-3-(4-hydroxyphenyl)acrylate (a), (*E*)-2-	S-40

isopropyl-5-methylphenyl-3-(4-hydroxyphenyl)acrylate (b) and (*E)*-2-acetyl-5-methoxyphenyl-3-(4-methoxyphenyl)acrylate (c).	
Figure S26. Docking of *(E*)-2-acetyl-5-methoxyphenyl-3-(4-hydroxyphenyl)acrylate to oxy-tyrosinase.	S-41
Figure S27. Docking of (*E*)-2-isopropyl-5-methylphenyl-3-(4-hydroxyphenyl)acrylate to oxy-tyrosinase.	S-42
S2.11. Figure S28. Chemical structure of stilbenes: Strebluses C (a) and Strebluses D (b).	S-43
Figure S29. Docking of Streblus C to oxy-tyrosinase.	S-44
Figure S30. Docking of Streblus D to oxy-tyrosinase.	S-45
S3. Tables.	S-46
S3.1. Table S1. PubChem CID numbers correspond to the structure used to build the corresponding ligands.	S-46
S3.2. Table S2. Variation of $\boldsymbol{i_D}$ to different $[\mathbf{I}]_\mathbf{0}$ and $[\mathbf{D}]_\mathbf{0} = \mathbf{K_m^D}$	S-48
S3.3. Table S3. $\boldsymbol{i_D}$ values for $[\mathbf{D}]_\mathbf{0} = \mathbf{2K_m^D}$ and $[\mathbf{D}]_\mathbf{0} = \mathbf{K_m^D}$	S-49
S3.4. Table S4. Ligand-protein interactions observed in the docking conformations of the oxy and met forms of tyrosinase in the cases of competitive inhibition.	S-50
S4. References.	S-54

S1. Kinetic analysis.

S1.1. Catalysis.

S1.1.1 Tyrosinase catalysis.

The equation for the initial rate of action of the enzyme on L-dopa (D) for the mechanism shown in Scheme 1 is:

$$V_0^{D,DC} = \frac{\alpha_1[O_2]_0[D]_0[E]_0}{2(\beta_0+\beta_1[D]_0+\beta_2[O_2]_0+\beta_3[O_2]_0[D]_0)} \quad \text{(S1)}$$

where DC is L-dopachrome.

Analytic expressions of α_1 y $\beta_0 - \beta_3$ are:

$$\alpha_1 = 2k_2k_3k_8k_6k_7$$

$$\beta_0 = k_2k_3k_{-8}(k_7 + k_{-6})$$

$$\beta_1 = k_2k_3k_6k_7 \quad \text{(S2)}$$

$$\beta_2 = k_8k_6k_7(k_{-2} + k_3) + k_2k_3k_8(k_7 + k_{-6})$$

$$\beta_3 = k_2k_8k_6(k_7 + k_3)$$

Considering that the concentration of oxygen is saturating $[O_2]_0 \to \infty$ [1,2] the equation (S1) can be simplified to:

$$V_0^{D,DC} = \frac{\alpha_1[D]_0[E]_0}{2(\beta_2+\beta_3[D]_0)} \quad \text{(S3)}$$

or depending on the kinetic parameters,

$$V_0^{D,DC} = \frac{V_{max}^{D,DC}[D]_0}{K_m^D+[D]_0} \quad \text{(S4)}$$

Taking into account that:

$$k_3 \gg k_7, k_7 \gg k_{-6}, k_3 \gg k_{-2} \quad \text{(S5)}$$

results:

$$V_{max}^{D,DC} = \frac{k_3k_7}{k_3+k_7}[E]_0 \cong k_7[E]_0 \quad \text{(S6)}$$

$$K_m^D \cong \frac{k_7}{k_6} \quad \text{(S7)}$$

S1.2. Inhibition.

S1.2.1. Tyrosinase inhibition.

S1.2.2. The rate equation in the presence of an inhibitor acting according to Scheme 2 would be:

$$V_{0,i}^{D,DC} = \frac{V_{max}^{D,DC}[D]_0}{K_m^D\left(1+\frac{[I]_0}{K_{I_1}^{app}}\right)+[D]_0\left(1+\frac{[I]_0}{K_{I_2}^{app}}\right)} \quad \text{(S8)}$$

with:

$$K_{I_1}^{app} = \frac{(k_2+k_6)K_{11}K_{14}}{k_2K_{11}+k_6K_{14}} \quad \text{(S9)}$$

and

$$K_{I_2}^{app} = \frac{(k_3+k_7)K_{12}K_{15}}{k_7K_{15}+k_3K_{12}} \quad \text{(S10)}$$

Inhibition types (Scheme 2):

- Competitive, with $K_{I_2}^{app} \to \infty$
- Non-competitive, with $K_{I_1}^{app} = K_{I_2}^{app}$
- Uncompetitive, with $K_{I_1}^{app} \to \infty$
- Mixed, with $K_{I_1}^{app} \neq K_{I_2}^{app}$

Thus, in the case of a competitive inhibitor:

$$V_{0,i}^{D,DC} = \frac{V_{max}^{D,DC}[D]_0}{K_m^D\left(1+\frac{[I]_0}{K_{I_1}^{app}}\right)+[D]_0} \quad \text{(S11)}$$

A non-competitive inhibitor:

$$V_{0,i}^{D,DC} = \frac{\frac{V_{max}^{D,DC}[D]_0}{1+\frac{[I]_0}{K_{I_2}^{app}}}}{K_m^D+[D]_0} \quad \text{(S12)}$$

A uncompetitive inhibitor:

$$V_{0,i}^{D,DC} = \frac{\frac{V_{max}^{D,DC}[D]_0}{1+\frac{[I]_0}{K_{I_2}^{app}}}}{\frac{K_m^D}{1+\frac{[I]_0}{K_{I_2}^{app}}}+[D]_0} \quad \text{(S13)}$$

The rate equation in the presence of a mixed inhibitor is expressed by equation (S8).

S1.2.3. Analytical expressions of the degree of inhibition as a function of $[I]_0$ and $[D]_0$ as well as a function of $[I]_0$ and n, where $n = [D]_0/K_m^D$.

The expression of the degree of inhibition in diphenolase activity is:

$$i_D = \frac{V_0^{D,DC} - V_{0,i}^{D,DC}}{V_0^{D,DC}} \times 100 \quad \text{(S14)}$$

Competitive inhibitor. The degree of inhibition is:

$$i_D^C = \frac{K_m^D\left(1+\frac{[I]_0}{K_{I_1}^{app}}\right)-K_m^D}{K_m^D\left(1+\frac{[I]_0}{K_{I_1}^{app}}\right)+[D]_0} \quad \text{(S15)}$$

If $[D]_0 = nK_m^D$, results:

$$i_D^C = \frac{[I]_0}{(1+n)K_{I_1}^{app}+[I]_0} \times 100 \quad \text{(S16)}$$

and

$$i_D^C = \frac{ab}{b+n} \quad \text{(S17)}$$

with:

$$a = \frac{[I]_0}{K_I^{app}+[I]_0} \text{x } 100 \quad \text{(S18)}$$

$$b = \frac{K_{I_1}^{app}+[I]_0}{K_{I_1}^{app}} \quad \text{(S19)}$$

Then:

$$IC_{50}^C = (1+n)K_{I_1}^{app} \quad \text{(S20)}$$

Non-competitive inhibitor. The degree of inhibition is:

$$i_{\mathrm{D}}^{\mathrm{NC}} = \frac{[\mathrm{I}]_0}{\mathrm{K}_{\mathrm{I}}^{\mathrm{app}}+[\mathrm{I}]_0} \mathrm{x}\ 100 \quad \text{(S21)}$$

and

$$\mathrm{IC}_{50}^{\mathrm{NC}} = \mathrm{K}_{\mathrm{I}}^{\mathrm{app}} \quad \text{(S22)}$$

Uncompetitive inhibitor. The degree of inhibition is:

$$i_{\mathrm{D}}^{\mathrm{U}} = \frac{\frac{[\mathrm{I}]_0}{\mathrm{K}_{\mathrm{I}_2}^{\mathrm{app}}}[\mathrm{D}]_0}{\mathrm{K}_{\mathrm{m}}^{\mathrm{D}}+\left(1+\frac{[\mathrm{I}]_0}{\mathrm{K}_{\mathrm{I}_2}^{\mathrm{app}}}\right)[\mathrm{D}]_0} \times 100 = \frac{\frac{[\mathrm{I}]_0 \mathrm{n}}{\left([\mathrm{I}]_0+\mathrm{K}_{\mathrm{I}_2}^{\mathrm{app}}\right)}}{\frac{\mathrm{K}_{\mathrm{I}_2}^{\mathrm{app}}}{\left([\mathrm{I}]_0+\mathrm{K}_{\mathrm{I}_2}^{\mathrm{app}}\right)}+\mathrm{n}} \times 100 \quad \text{(S23)}$$

Therefore:

$$i_{\mathrm{D}}^{\mathrm{U}} = \frac{[\mathrm{I}]_0}{\mathrm{K}_{\mathrm{I}_2}^{\mathrm{app}}\left(1+\frac{1}{\mathrm{n}}\right)+[\mathrm{I}]_0} \times 100 \quad \text{(S24)}$$

and

$$i_{\mathrm{D}}^{\mathrm{U}} = \frac{\mathrm{an}}{\mathrm{b+n}} \quad \text{(S25)}$$

with:

$$\mathrm{a} = \frac{[\mathrm{I}]_0}{\mathrm{K}_{\mathrm{I}_2}^{\mathrm{app}}+[\mathrm{I}]_0} \mathrm{x}\ 100 \quad \text{(S26)}$$

$$\mathrm{b} = \frac{\mathrm{K}_{\mathrm{I}_2}^{\mathrm{app}}}{\mathrm{K}_{\mathrm{I}_2}^{\mathrm{app}}+[\mathrm{I}]_0} \quad \text{(S27)}$$

Then,

$$\mathrm{IC}_{50}^{\mathrm{U}} = \left(1+\frac{1}{\mathrm{n}}\right)\mathrm{K}_{\mathrm{I}_2}^{\mathrm{app}} \quad \text{(S28)}$$

Mixed inhibition:

The rate equation is expressed in equation (S8).

The degree of inhibition is:

$$i_{\mathrm{D}}^{\mathrm{M}} = \frac{\mathrm{K}_{\mathrm{m}}^{\mathrm{D}}\frac{[\mathrm{I}]_0}{\mathrm{K}_{\mathrm{I}_1}^{\mathrm{app}}}+\frac{[\mathrm{I}]_0}{\mathrm{K}_{\mathrm{I}_2}^{\mathrm{app}}}[\mathrm{D}]_0}{\mathrm{K}_{\mathrm{m}}^{\mathrm{D}}\left(1+\frac{[\mathrm{I}]_0}{\mathrm{K}_{\mathrm{I}_1}^{\mathrm{app}}}\right)+\left(1+\frac{[\mathrm{I}]_0}{\mathrm{K}_{\mathrm{I}_2}^{\mathrm{app}}}\right)[\mathrm{D}]_0} \times 100 \quad \text{(S29)}$$

According to inhibitor concentration:

$$i_D^M = \frac{[I]_0}{\frac{K_{I_1}^{app}K_{I_2}^{app}(1+n)}{K_{I_1}^{app}+nK_{I_2}^{app}}+[I]_0} \times 100 \tag{S30}$$

and

$$i_D^M = \frac{a+bn}{c+n} \tag{S31}$$

with:

$$a = \frac{[I]_0 K_{I_2}^{app}}{\left(K_{I_2}^{app}+[I]_0\right)K_{I_1}^{app}} \text{ x } 100 \tag{S32}$$

$$b = \frac{[I]_0}{K_{I_2}^{app}+[I]_0} \text{ x } 100 \tag{S33}$$

$$c = \frac{\left(K_{I_1}^{app}+[I]_0\right)K_{I_2}^{app}}{\left(K_{I_2}^{app}+[I]_0\right)K_{I_1}^{app}} \tag{S34}$$

Thus, we obtain:

$$IC_{50}^M = \frac{K_{I_1}^{app}K_{I_2}^{app}(1+n)}{K_{I_2}^{app}+nK_{I_1}^{app}} \tag{S35}$$

S1.3. Differential equations for the different inhibition mechanisms.

S1.3.1. Competitive inhibition:

$$[\dot{\mathrm{Em}}] = \mathrm{k_{-2}[EmD] + k_7[EoxD] + k_{-11}[EmI] - k_2[Em][D] - k_{11}[I][Em]}$$

$$[\dot{\mathrm{EmI}}] = \mathrm{k_{11}[I][Em] - k_{-11}[EmI]}$$

$$[\dot{\mathrm{EmD}}] = \mathrm{k_2[D][Em] - k_{-2}[EmD] - k_3[EmD]}$$

$$[\dot{\mathrm{Ed}}] = \mathrm{k_3[EmD] + k_{-8}[Eox] - k_8[B][Ed]}$$

$$[\dot{\mathrm{EoxD}}] = \mathrm{k_6[D][Eox] - k_{-6}[EoxD] - k_7[EoxD]}$$

$$[\dot{\mathrm{Eox}}] = \mathrm{k_8[B][Ed] + k_{-6}[EoxD] + k_{-14}[EoxI] - k_{-8}[Eox] - k_6[D][Eox] - k_{14}[I][Eox]}$$

$$[\dot{\mathrm{EoxI}}] = \mathrm{k_{14}[I][Eox] - k_{-14}[EoxI]}$$

$$[\dot{\mathrm{Q}}] = \mathrm{k_3[EmD] + k_7[EoxD] - k_{16}[Q]}$$

$$[\dot{\mathrm{DC}}] = \mathrm{0.5k_{16}[Q]}$$

$$[\dot{\mathrm{D}}] = \mathrm{k_{-2}[EmD] - k_2[Em][D] + k_{-6}[EoxD] - k_6[Eox][D] + 0.5k_{16}[Q]}$$

$$[\dot{\mathrm{B}}] = 0$$

$$[\dot{\mathrm{I}}] = 0$$

S1.3.2. Non-competitive inhibition:

The values of the apparent inhibition constants must comply $\mathrm{K_{I_1}^{app} = K_{I_2}^{app}}$.

$$[\dot{\mathrm{Em}}] = \mathrm{k_{-2}[EmD] + k_7[EoxD] + k_{-11}[EmI] - k_2[Em][D] - k_{11}[I][Em]}$$

$$[\dot{\mathrm{EmI}}] = \mathrm{k_{11}[I][Em] - k_{-11}[EmI]}$$

$$[\dot{\mathrm{EmD}}] = \mathrm{k_2[D][Em] + k_{-12}[EmDI] - k_{-2}[EmD] - k_3[EmD] - k_{12}[I][EmD]}$$

$$[\dot{\mathrm{EmDI}}] = \mathrm{k_{12}[I][EmD] - k_{-12}[EmDI]}$$

$$[\dot{\mathrm{Ed}}] = \mathrm{k_3[EmD] + k_{-8}[Eox] - k_8[B][Ed]}$$

$$[\dot{\mathrm{EoxD}}] = \mathrm{k_6[D][Eox] + k_{-15}[EoxDI] - k_{-6}[EoxD] - k_7[EoxD] - k_{15}[I][EoxD]}$$

$[\dot{Eox}] = k_8[B][Ed] + k_{-6}[EoxD] + k_{-14}[EoxI] - k_{-8}[Eox] - k_6[D][Eox] - k_{14}[I][Eox]$

$[\dot{EoxI}] = k_{14}[I][Eox] - k_{-14}[EoxI]$

$[\dot{EoxDI}] = k_{15}[I][EoxD] - k_{-15}[EoxDI]$

$[\dot{Q}] = k_3[EmD] + k_7[EoxD] - k_{16}[Q]$

$[\dot{DC}] = 0.5k_{16}[Q]$

$[\dot{D}] = k_{-2}[EmD] - k_2[Em][D] + k_{-6}[EoxD] - k_6[Eox][D] + 0.5k_{16}[Q]$

$[\dot{B}] = 0$

$[\dot{I}] = 0$

S1.3.3. Uncompetitive inhibition:

$[\dot{Em}] = k_{-2}[EmD] + k_7[EoxD] - k_2[Em][D]$

$[\dot{EmDI}] = k_{12}[I][EmD] - k_{-12}[EmDI]$

$[\dot{EmD}] = k_2[D][Em] + k_{-12}[EmDI] - k_{-2}[EmD] - k_{12}[EmD][I] - k_3[EmD]$

$[\dot{Ed}] = k_3[EmD] + k_{-8}[Eox] - k_8[B][Ed]$

$[\dot{EoxD}] = k_6[D][Eox] + k_{-15}[EoxDI] - k_{-6}[EoxD] - k_7[EoxD] - k_{15}[EoxD][I]$

$[\dot{Eox}] = k_8[B][Ed] + k_{-6}[EoxD] - k_{-8}[Eox] - k_6[D][Eox]$

$[\dot{EoxDI}] = k_{15}[I][EoxD] - k_{-15}[EoxDI]$

$[\dot{Q}] = k_3[EmD] + k_7[EoxD] - k_{16}[Q]$

$[\dot{DC}] = 0.5k_{16}[Q]$

$[\dot{D}] = k_{-2}[EmD] - k_2[Em][D] + k_{-6}[EoxD] - k_6[Eox][D] + 0.5k_{16}[Q]$

$[\dot{B}] = 0$

$[\dot{I}] = 0$

S1.3.4. Mixed inhibition:

The equations are those corresponding to the non-competitive inhibitor, but in this case, $K_{I_1}^{app} \neq K_{I_2}^{app}$. In the case of mixed inhibitor type (1), $K_{I_1}^{app} > K_{I_2}^{app}$ and in the case of type (2), $K_{I_1}^{app} < K_{I_2}^{app}$.

$$[\dot{Em}] = k_{-2}[EmD] + k_7[EoxD] + k_{-11}[EmI] - k_2[Em][D] - k_{11}[I][Em]$$

$$[\dot{EmI}] = k_{11}[I][Em] - k_{-11}[EmI]$$

$$[\dot{EmD}] = k_2[D][Em] + k_{-12}[EmDI] - k_{-2}[EmD] - k_3[EmD] - k_{12}[I][EmD]$$

$$[\dot{EmDI}] = k_{12}[I][EmD] - k_{-12}[EmDI]$$

$$[\dot{Ed}] = k_3[EmD] + k_{-8}[Eox] - k_8[B][Ed]$$

$$[\dot{EoxD}] = k_6[D][Eox] + k_{-15}[EoxDI] - k_{-6}[EoxD] - k_7[EoxD] - k_{15}[I][EoxD]$$

$$[\dot{Eox}] = k_8[B][Ed] + k_{-6}[EoxD] + k_{-14}[EoxI] - k_{-8}[Eox] - k_6[D][Eox] - k_{14}[I][Eox]$$

$$[\dot{EoxI}] = k_{14}[I][Eox] - k_{-14}[EoxI]$$

$$[\dot{EoxDI}] = k_{15}[I][EoxD] - k_{-15}[EoxDI]$$

$$[\dot{Q}] = k_3[EmD] + k_7[EoxD] - k_{16}[Q]$$

$$[\dot{DC}] = 0.5k_{16}[Q]$$

$$[\dot{D}] = k_{-2}[EmD] - k_2[Em][D] + k_{-6}[EoxD] - k_6[Eox][D] + 0.5k_{16}[Q]$$

$$[\dot{B}] = 0$$

$$[\dot{I}] = 0$$

S1.4. Schemes.

S1.4.1. Scheme S1.

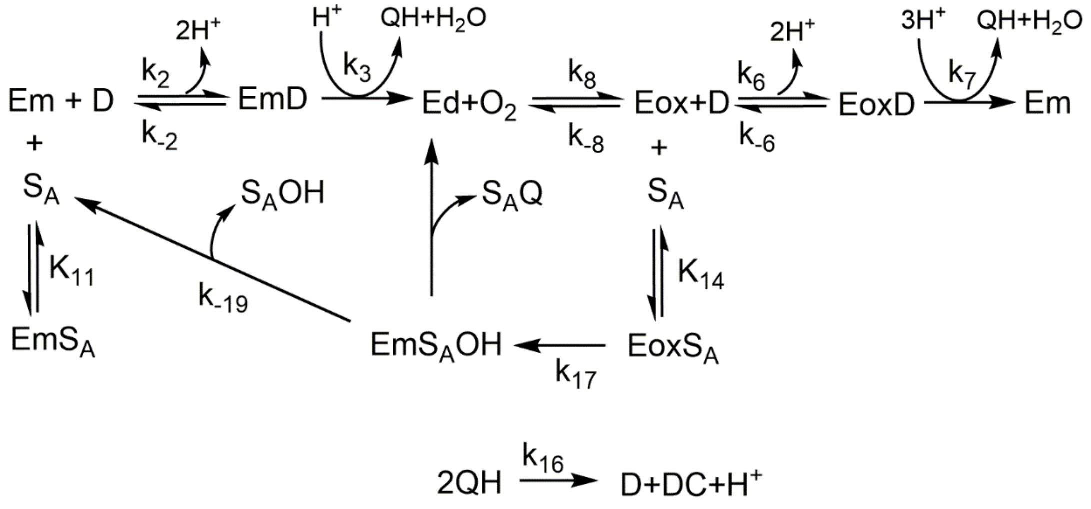

Scheme S1. Mechanism of action of tyrosinase on L-dopa in the presence of an alternative substrate (monophenol). Where: S_A is the alternative substrate, S_AOH is the hydroxylated alternative substrate, S_AQ is the o-quinone of the hydroxylated alternative substrate, EmS_A is the complex of Em with S_A, EmS_AOH is the complex of Em and hydroxylated S_A.

S1.4.2. Scheme S2.

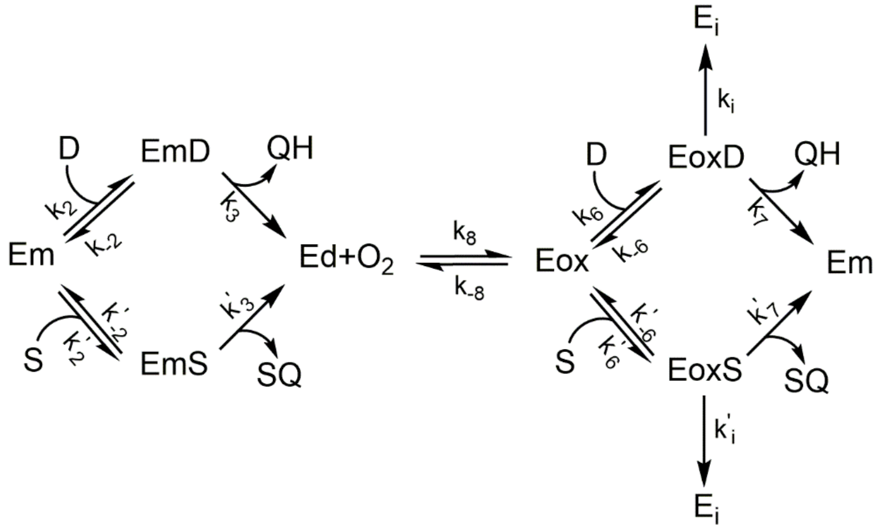

Scheme S2. Mechanism of action of tyrosinase on L-dopa (D) in the presence of a suicide substrate (*o*-diphenol). Where S is the suicide substrate, SQ is the *o*-quinone of the suicide substrate, EmS is the complex of Em with S, EoxS is the complex of Eox with S, and Ei is the inactive enzyme. L-dopa (*o*-diphenol) is also described as a suicide substrate.

S2. Chemical structures of the inhibitors described in the different figures. Docking of some of these molecules to oxy-tyrosinase or met-tyrosinase.

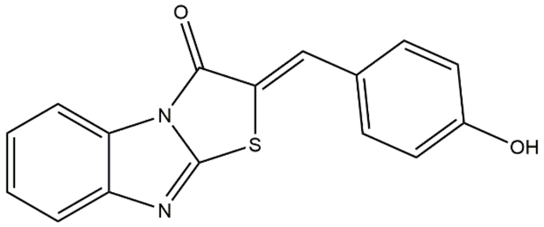

a

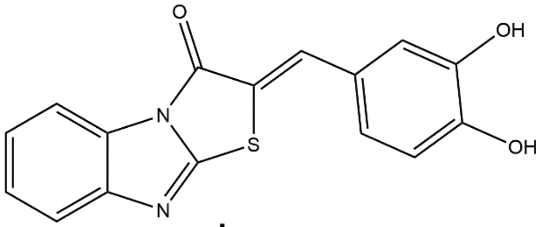

b

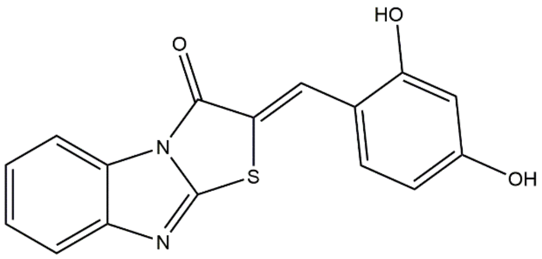

c

S2.1. **Figure S1**. Chemical structures of benzimidazothiazolone derivatives: (Z)-2-(4-Hydroxybenzylidene)benzo[4,5]imidazo[2,1-*b*]thiazol-3(2*H*)-one (a); (Z)-2-(3,4-Dihydroxybenzylidene)benzo[4,5]imidazo[2,1-*b*]thiazol-3(2*H*)-one (b); (Z)-2-(2,4-Dihydroxybenzylidene)benzo[4,5]imidazo[2,1-*b*]thiazol-3(2*H*)-one (c) [3].

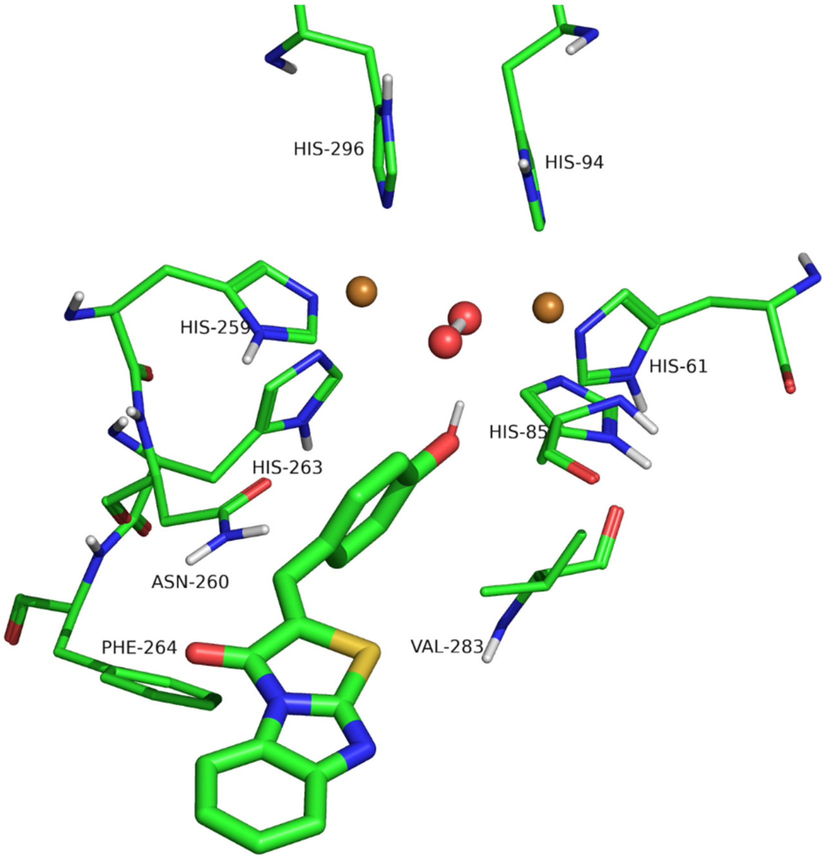

Figure S2. Docking of (Z)-2-(4-Hydroxybenzylidene)benzo[4,5]imidazo[2,1-*b*]thiazol-3(2*H*)-one to oxy-tyrosinase. Color scheme is as in Figure 3.

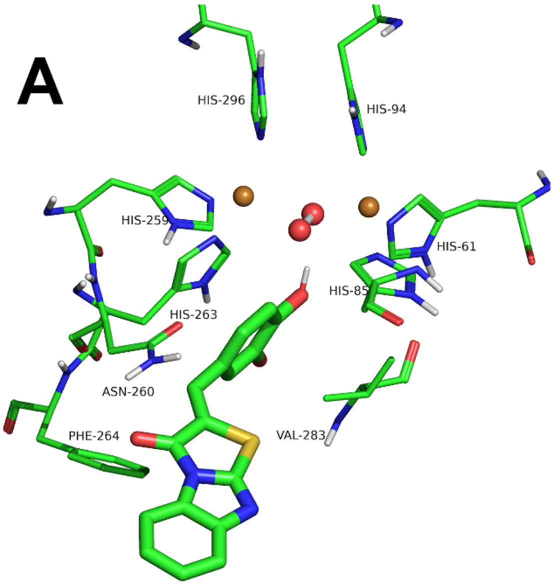

Figure S3. Docking of (Z)-2-(3,4-Dihydroxybenzylidene)benzo[4,5]imidazo[2,1-*b*]thiazol-3(2*H*)-one to oxy-tyrosinase **(A)** and met-tyrosinase **(B).** Color scheme is as in Figure 3.

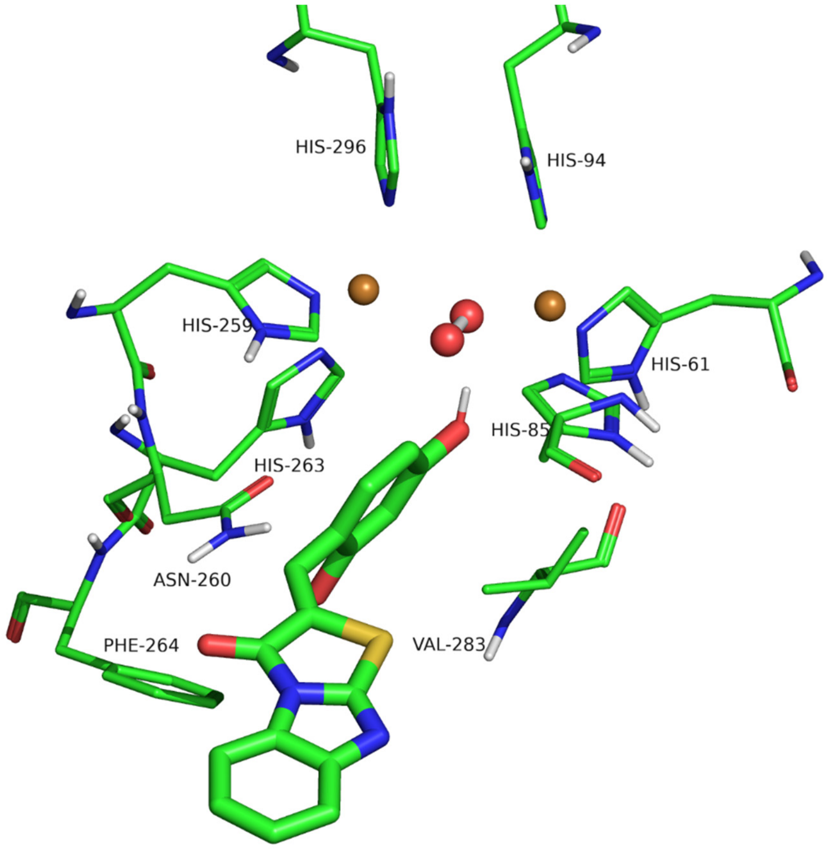

Figure S4. Docking of (Z)-2-(2,4-Dihydroxybenzylidene)benzo[4,5]imidazo[2,1-*b*]thiazol-3(2*H*)-one to oxy-tyrosinase. Color scheme is as in Figure 3.

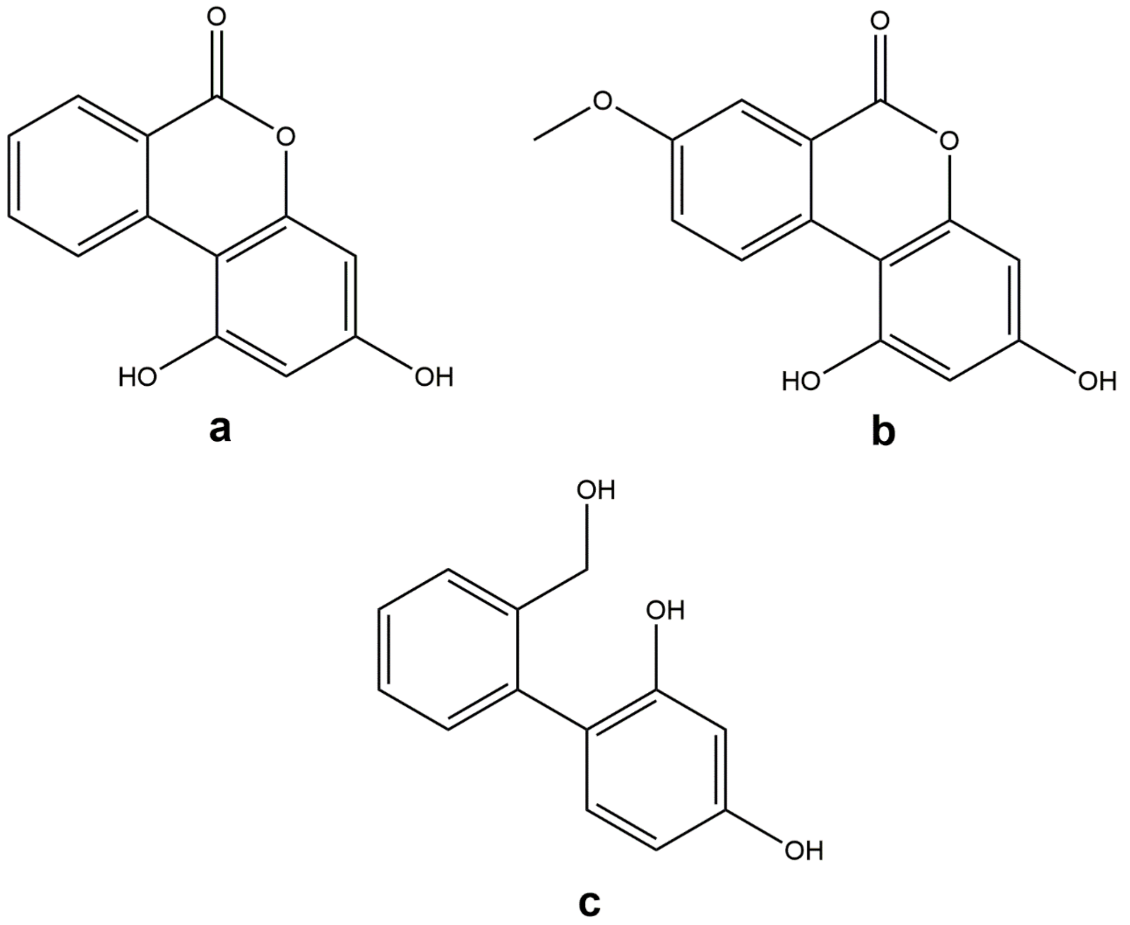

S2.2. **Figure S5.** Chemical structures of urolithin derivatives: 1,3-Dihydroxy-6*H*-benzo[c]chromen-6-one (a); 1,3-Dihydroxy-8-methoxy-6*H*-benzo[c]chromen-6-one (b) and 2’-(Hydroxymethyl)-[1,1’-biphenyl]-2,4-diol (c) [4].

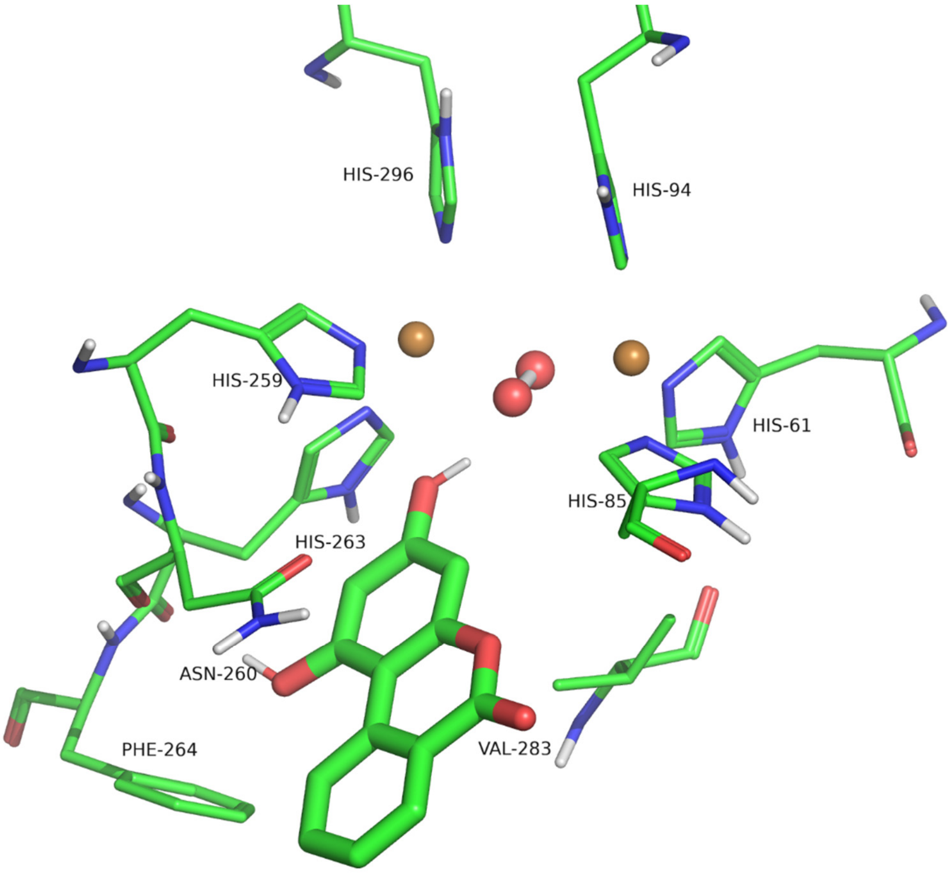

Figure S6. Docking of 1,3-Dihydroxy-6*H*-benzo[c]chromen-6-one to *oxy*-tyrosinase. Color scheme is as in Figure 3.

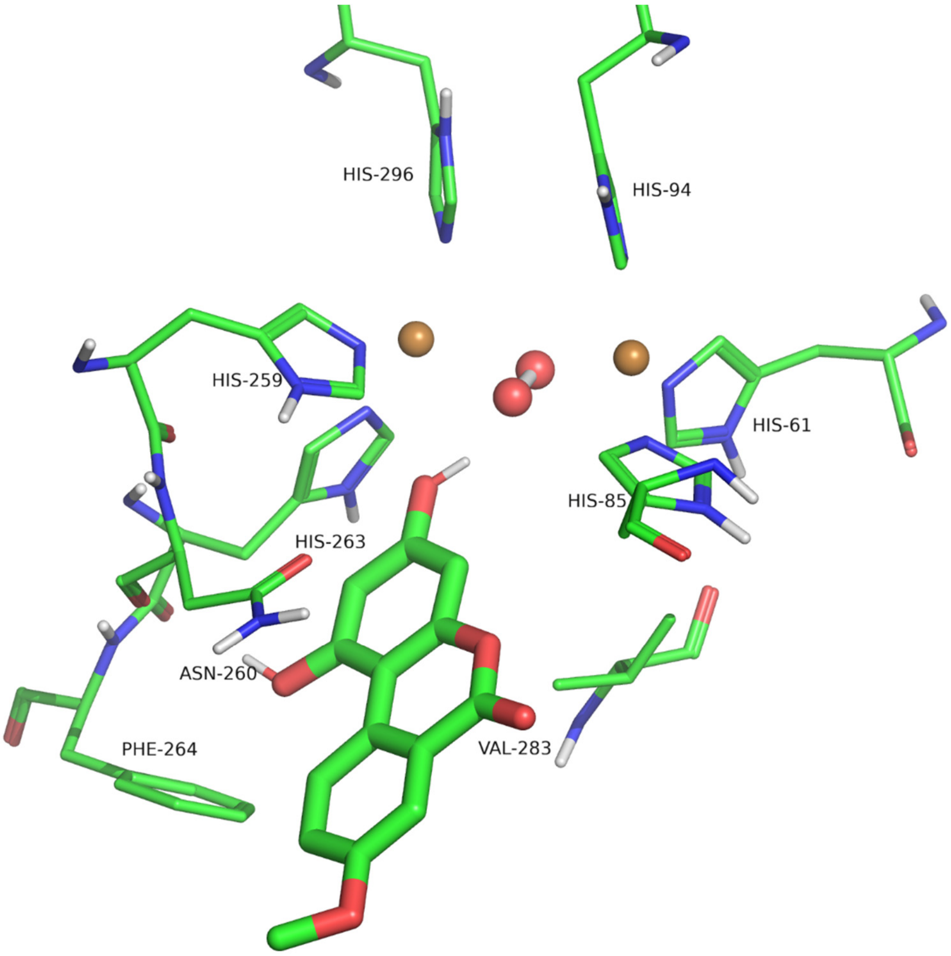

Figure S7. Docking of 1,3-Dihydroxy-8-methoxy-6*H*-benzo[c]chromen-6-one to *oxy*-tyrosinase. Color scheme is as in Figure 3.

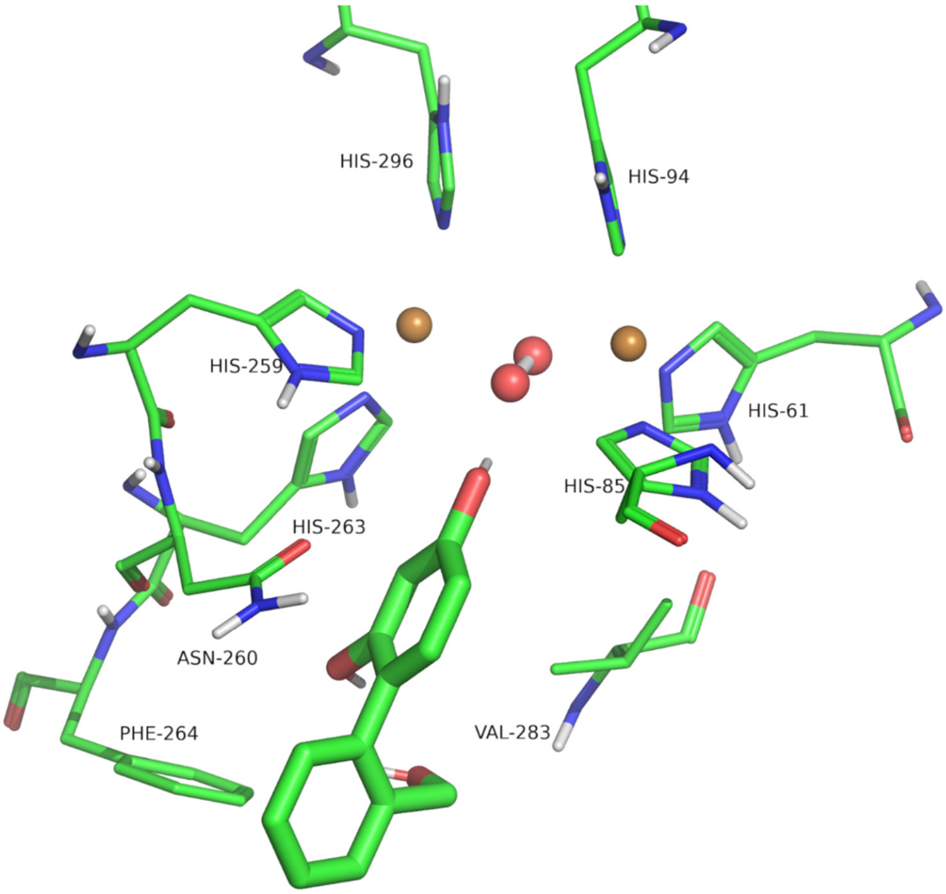

Figure S8. Docking of 2'-(Hydroxymethyl)-[1,1'-biphenyl]-2,4-diol to *oxy*-tyrosinase. Color scheme is as in Figure 3.

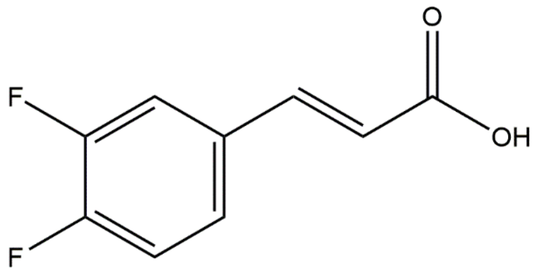

S2.3. **Figure S9.** Chemical structure of a cinnamic acid derivative: Trans-3,4-diflurocinnamic acid [5].

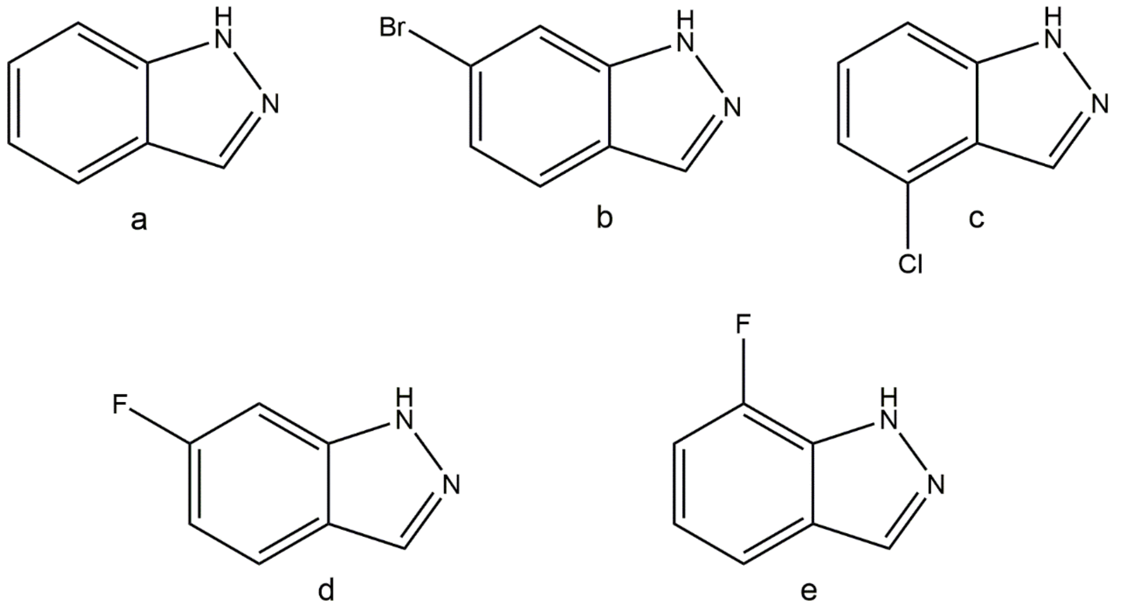

S2.4. **Figure S10.** Chemical structure of some indazoles: 7-bromo-1*H*-indazole (a); 6-bromo-1*H*-indazole (b); 4-chloro-1*H*-indazole (c); 6-fluoro-1*H*-indazole (d); 7-fluoro-1*H*-indazole (e) [6].

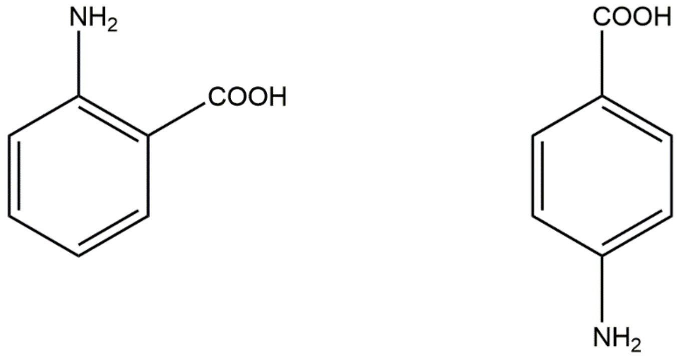

a **b**

S2.5. **Figure S11.** Chemical structure of benzoic acid derivatives: 2-amino benzoic acid (a); 4-amino benzoic acid (b) [7].

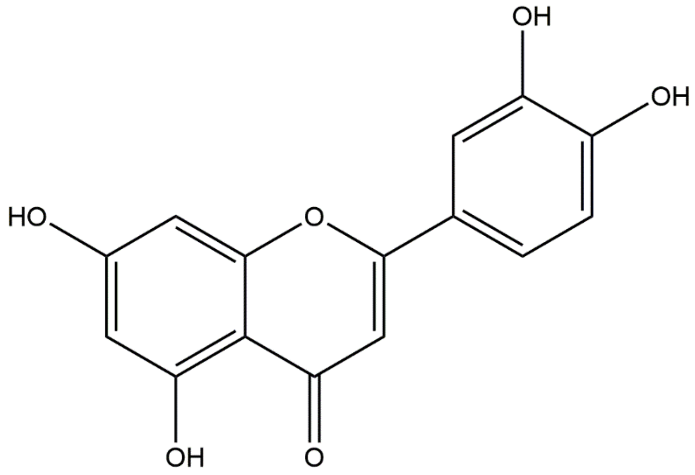

S2.6. **Figure S12.** Chemical structure of luteolin [8].

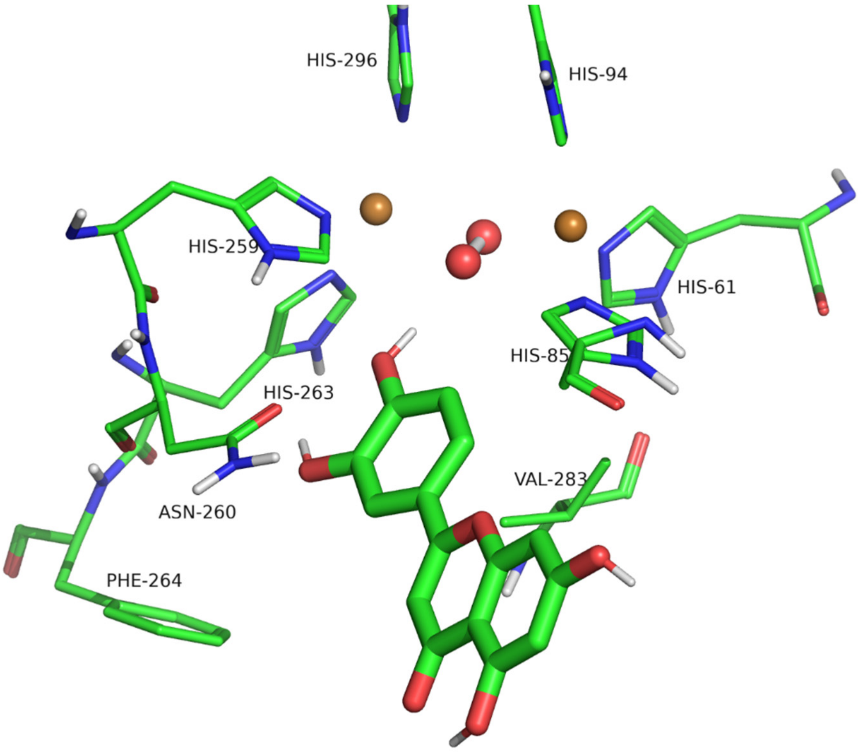

Figure S13. Docking of luteolin to oxy-tyrosinase. Color scheme is as in Figure 3.

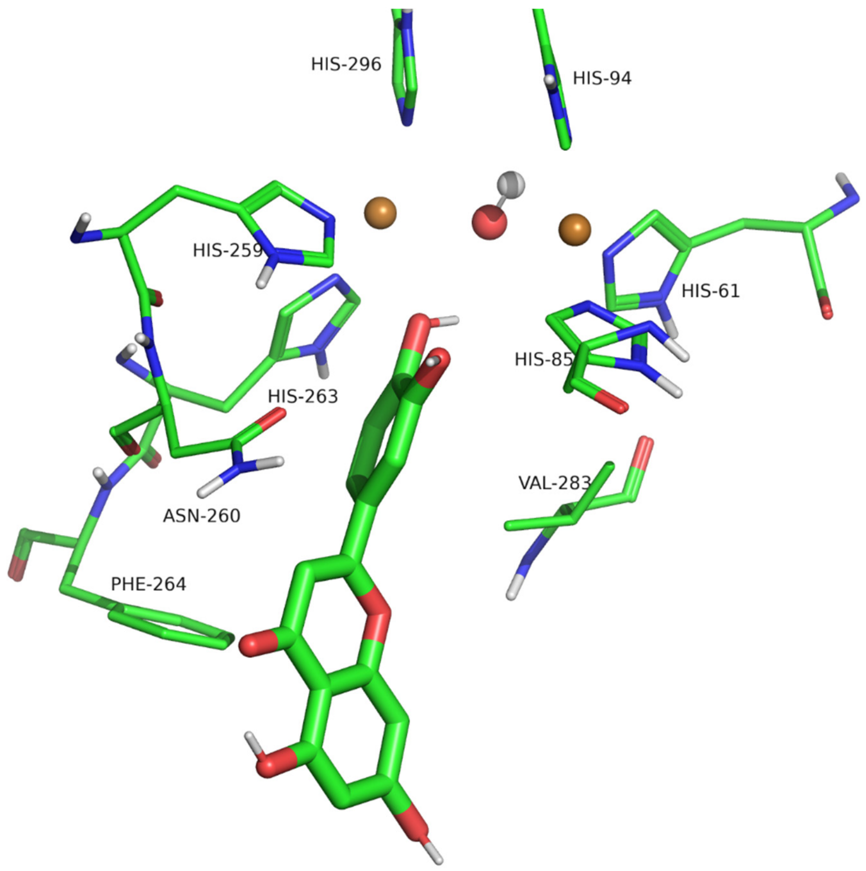

Figure S14. Docking of luteolin to met-tyrosinase. Colour scheme is as in Figure 3.

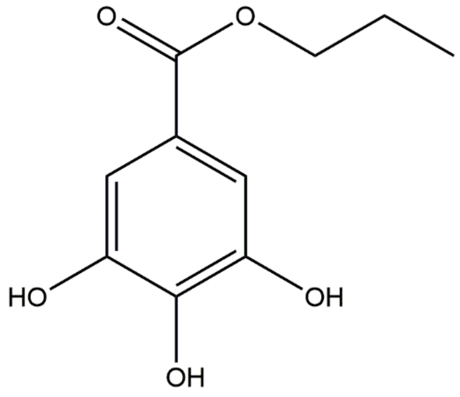

S2.7. **Figure S15.** Chemical structure of propyl gallate [9].

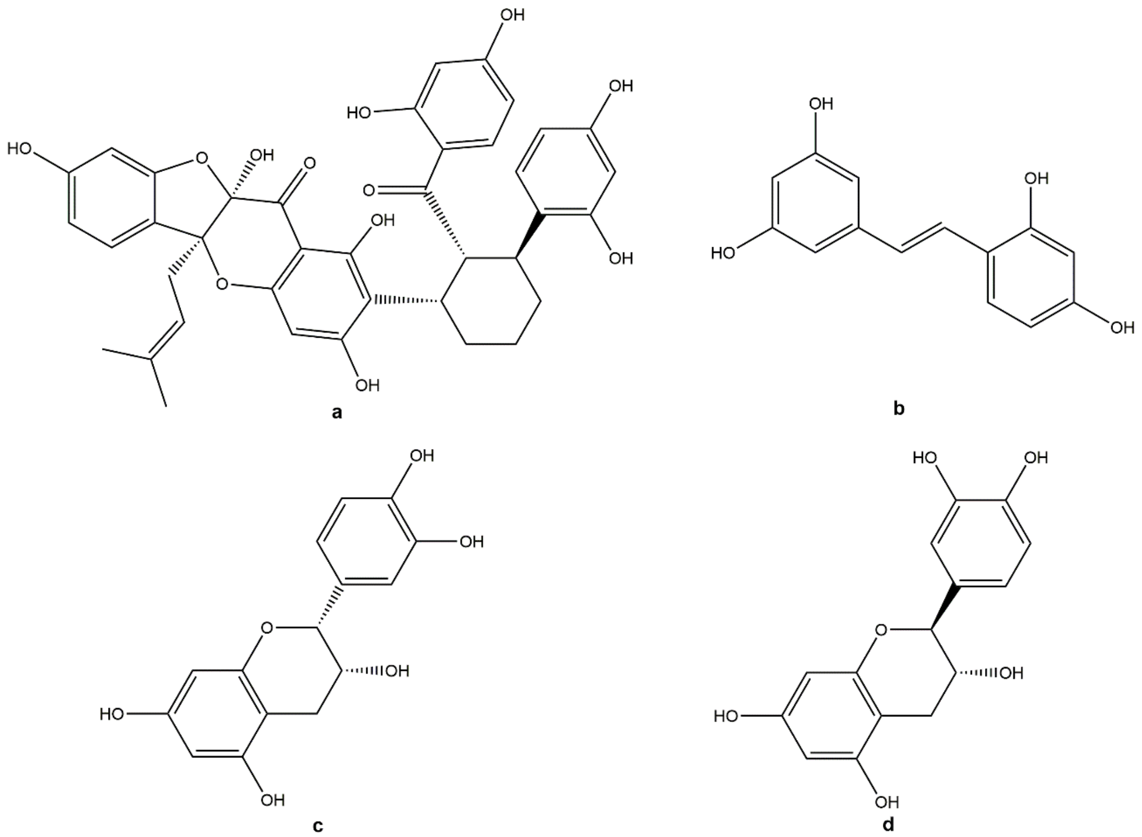

S2.8. **Figure S16.** Chemical structure of polyphenols: sanggenone C (a); oxyresveratrol (b); L-epicatechin (c) and catechin (d) [10].

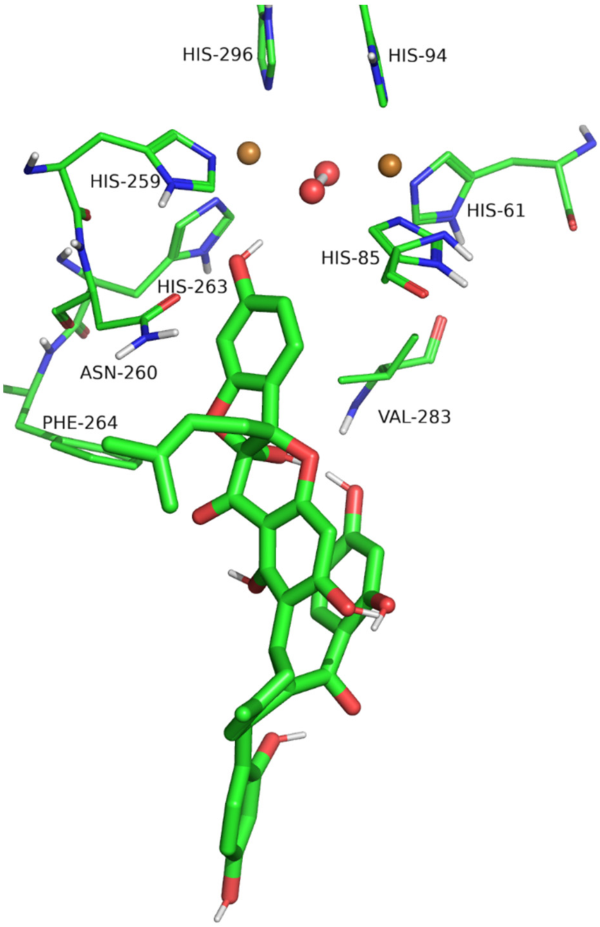

Figure S17. Docking of sanggenone C to oxy-tyrosinase. Color scheme is as in Figure 3.

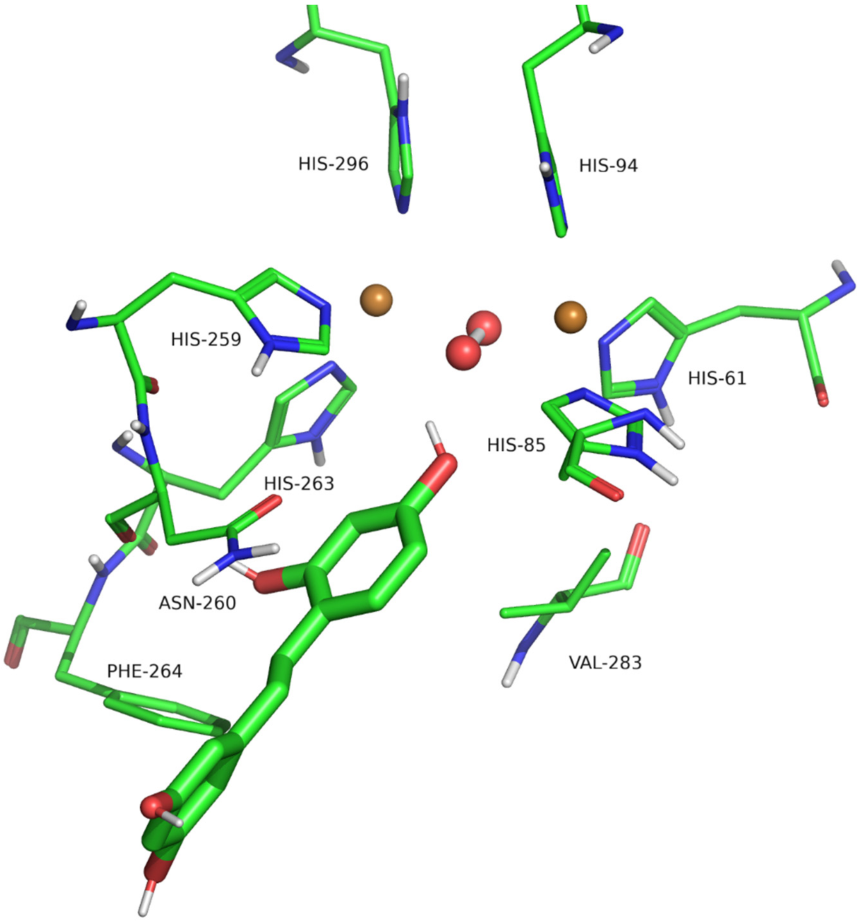

Figure S18. Docking of oxyresveratrol to oxy-tyrosinase. Color scheme is as in Figure 3.

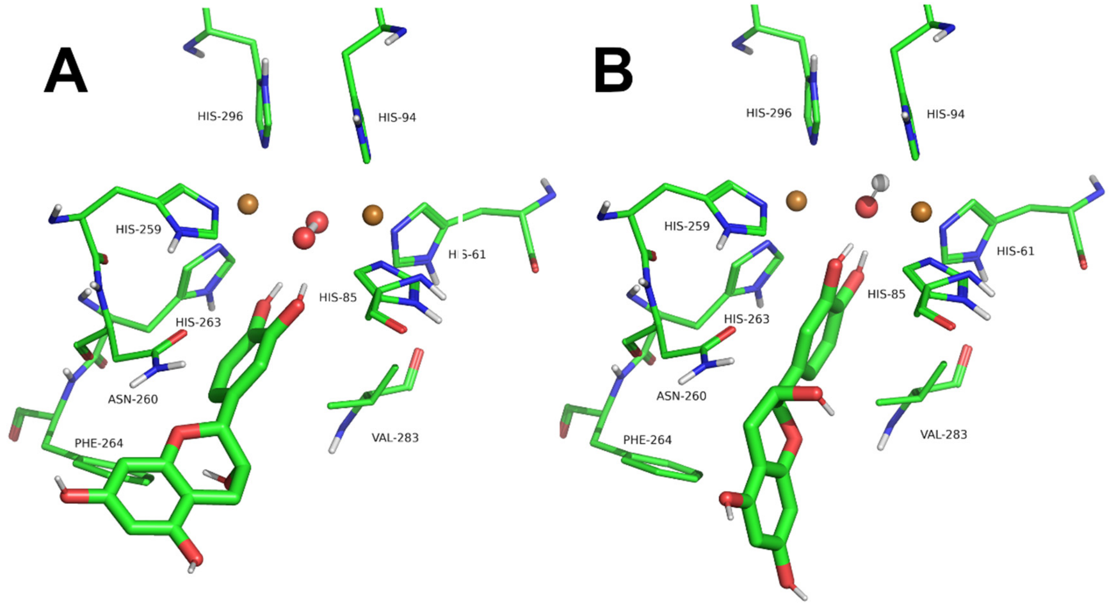

Figure S19A,B. Docking of L-epicatechin to oxy-tyrosinase **(A)** and met-tyrosinase **(B).** Color scheme is as in Figure 3.

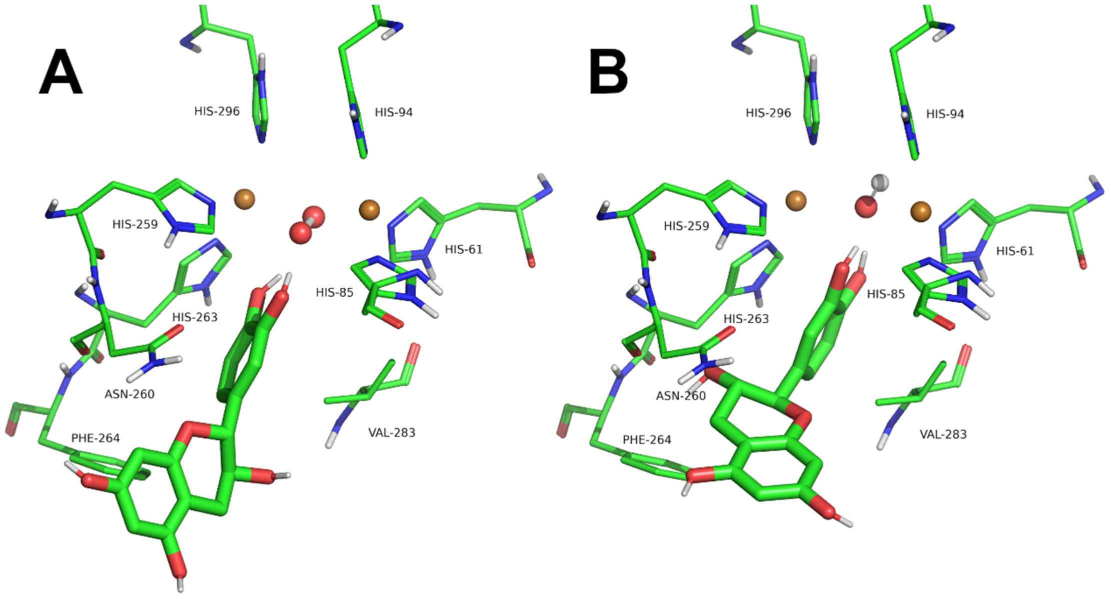

Figure S20A,B. Docking of catechin to oxy-tyrosinase **(A)** and met-tyrosinase **(B).** Color scheme is as in Figure 3.

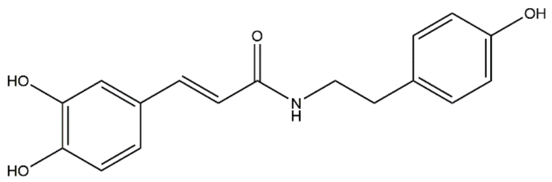

a

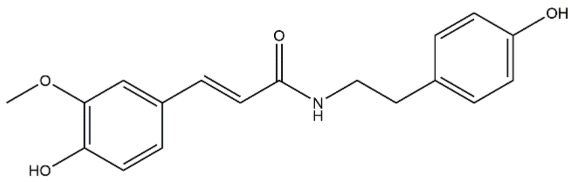

b

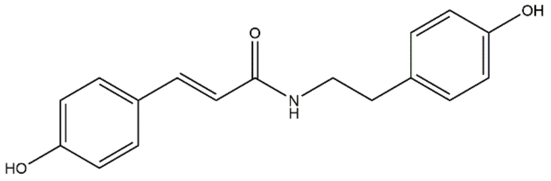

c

S2.9. **Figure S21.** Chemical structure of phenetyl cinnamamide derivatives: *N-trans*-caffeoyltyramine (a); *N-trans*-feruloyltyramine (b) and *N-trans*-coumaroyltyramine (c) [11].

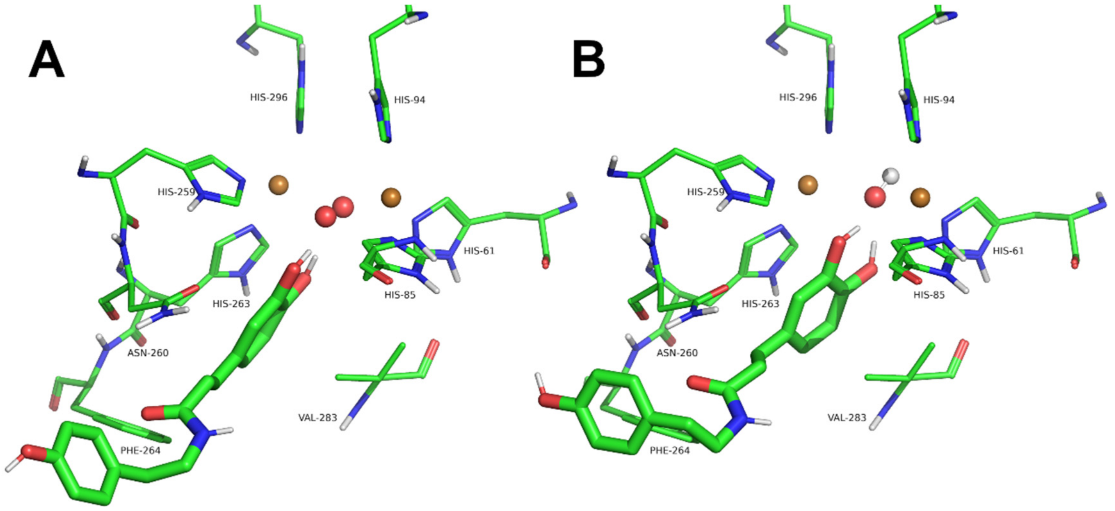

Figure S22A,B. Docking of *N-trans*-caffeoyltyramine to oxy-tyrosinase **(A)** and met-tyrosinase **(B).** Color scheme is as in Figure 3.

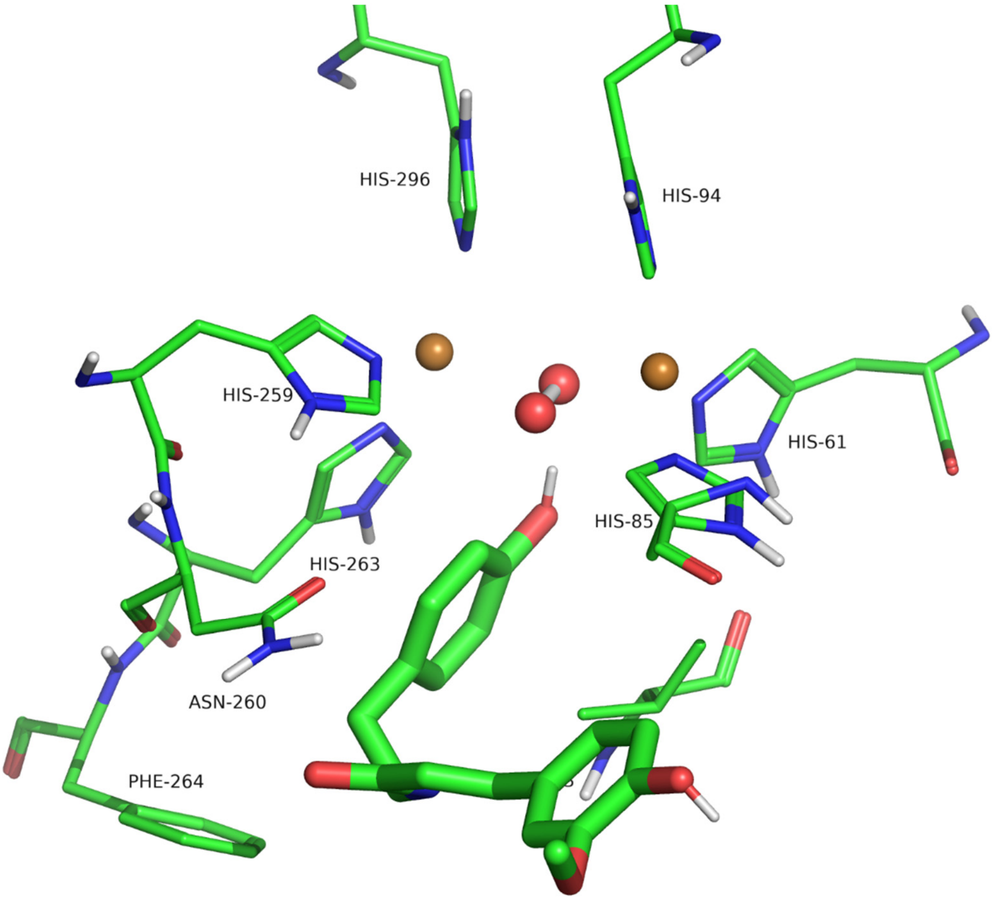

Figure S23. Docking of *N-trans*-feruloyltyramine to oxy-tyrosinase. Color scheme is as in Figure 3.

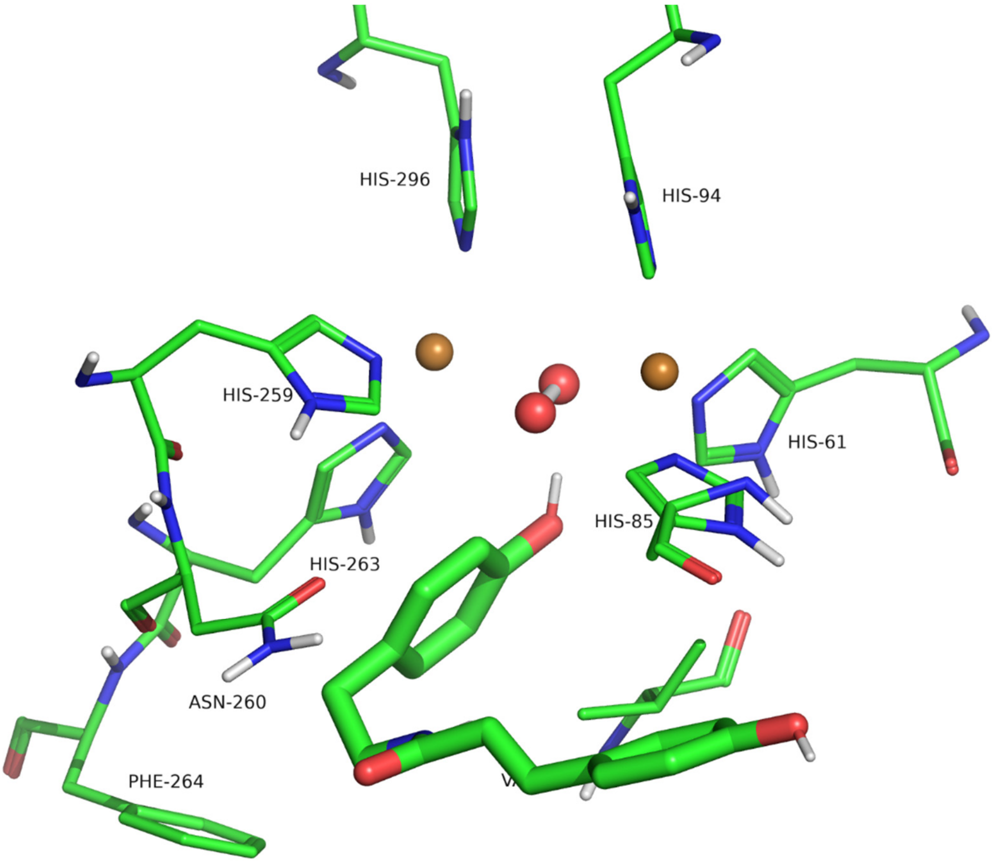

Figure S24. Docking of *N*-*trans*-coumaroyltyramine to oxy-tyrosinase. Color scheme is as in Figure 3.

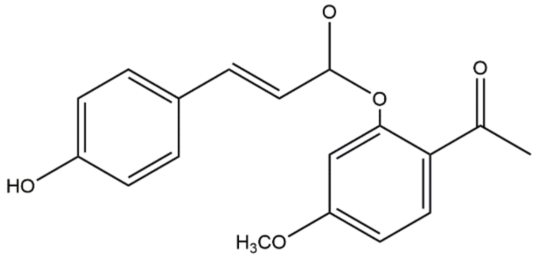

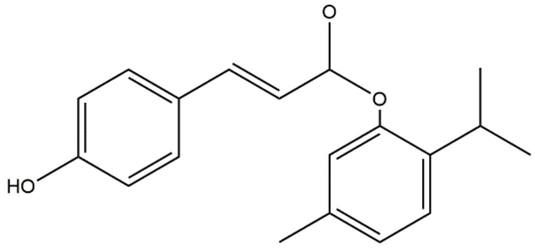

a **b**

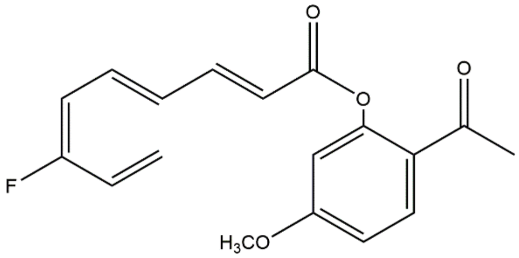

c

S2.10. **Figure S25.** Chemical structure of cinnamic acid ester derivatives: (*E*)-2-acetyl-5-methoxyphenyl-3-(4-hydroxyphenyl)acrylate (a), (*E*)-2-isopropyl-5-methylphenyl-3-(4-hydroxyphenyl)acrylate (b) and (*E)*-2-acetyl-5-methoxyphenyl-3-(4-methoxyphenyl)acrylate (c) [12].

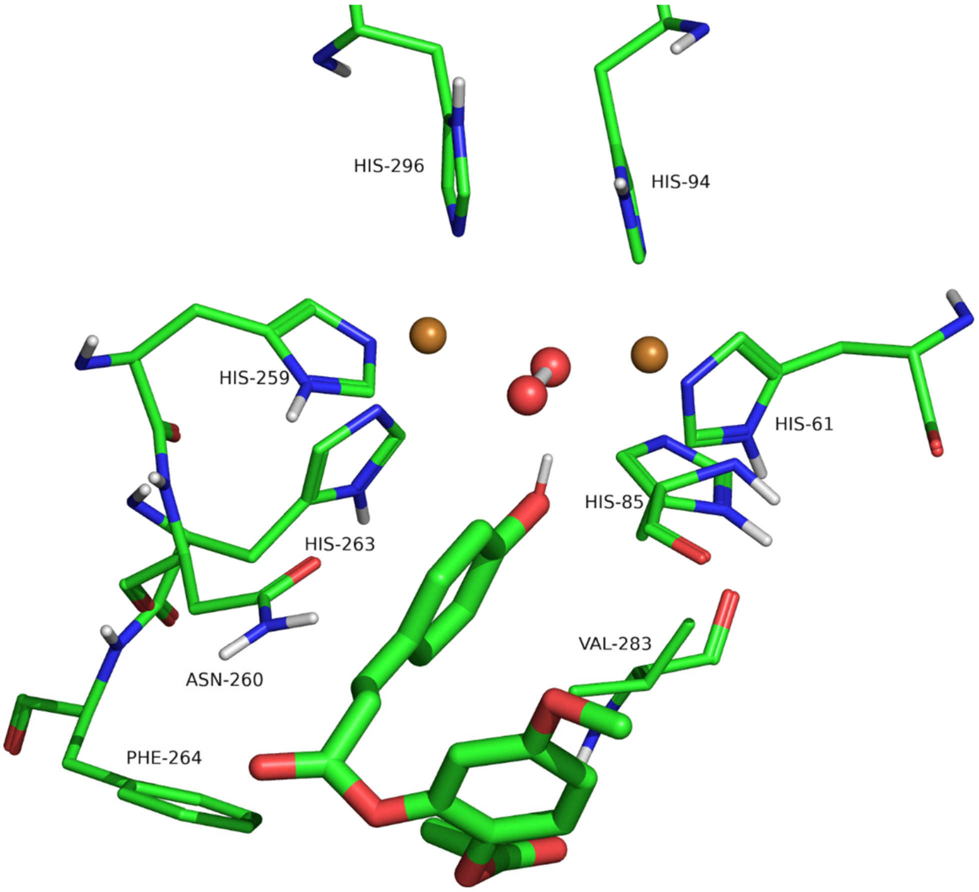

Figure S26. Docking of (*E*)-2-acetyl-5-methoxyphenyl-3-(4-hydroxyphenyl)acrylate to oxy-tyrosinase. Color scheme is as in Figure 3.

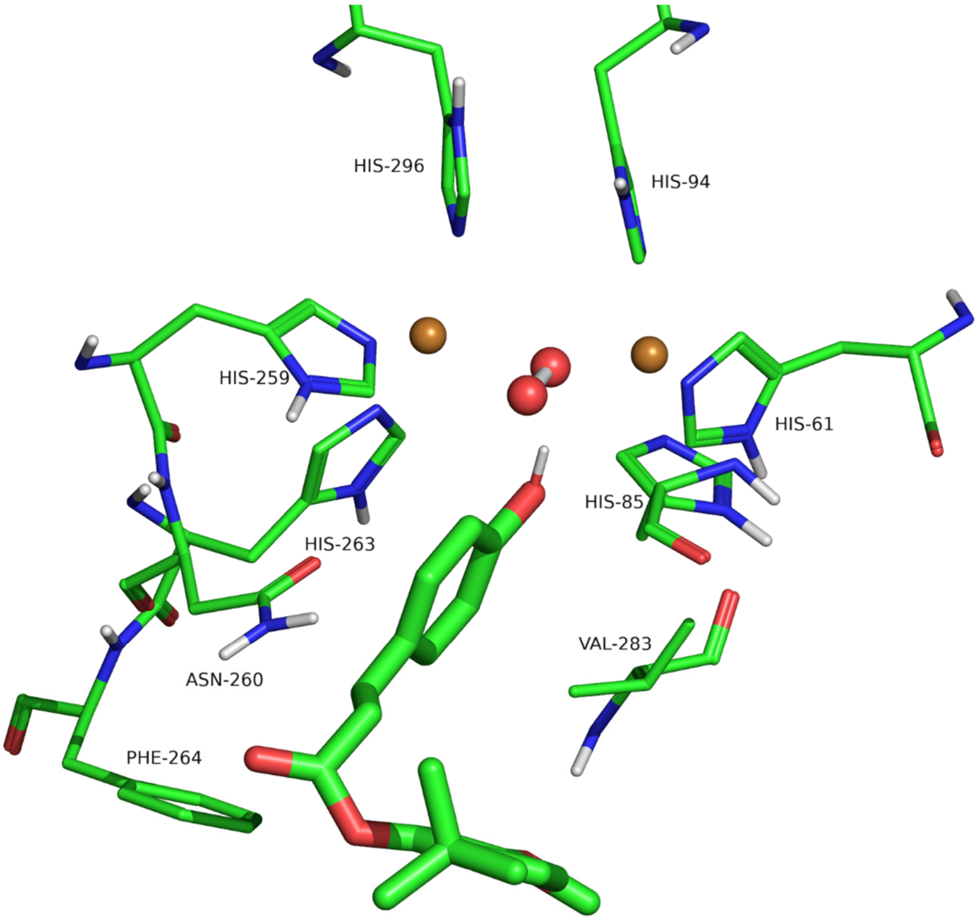

Figure S27. Docking of (*E*)-2-isopropyl-5-methylphenyl-3-(4-hydroxyphenyl)acrylate to oxy-tyrosinase. Color scheme is as in Figure 3.

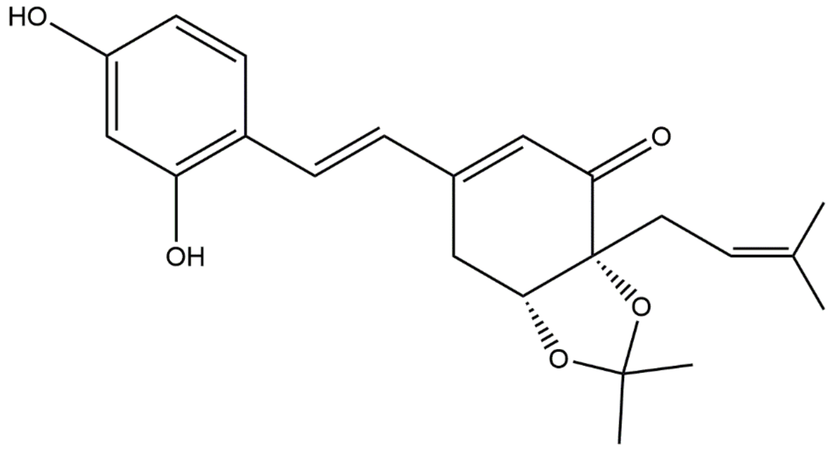

a

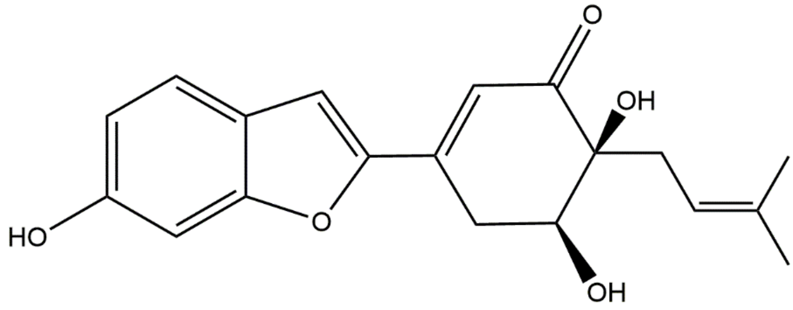

b

S2.11. **Figure S28.** Chemical structure of stilbenes: Strebluses C (a) and Strebluses D (b) [13].

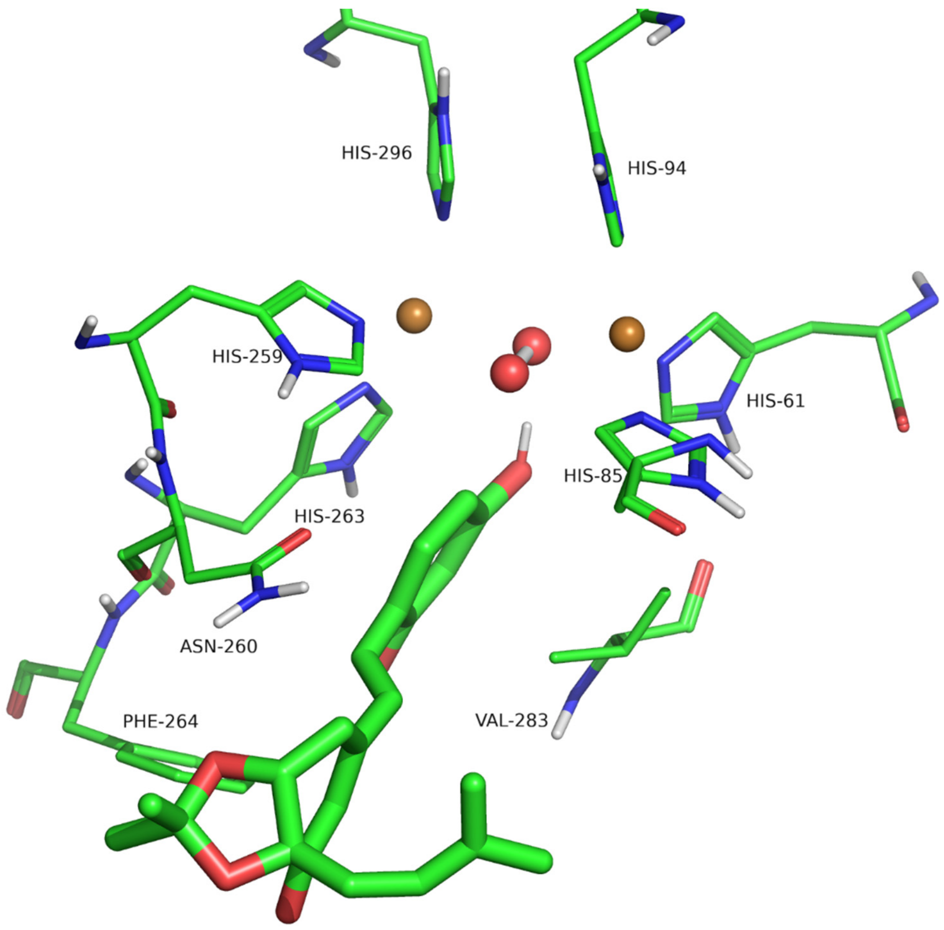

Figure S29. Docking of Streblus C to oxy-tyrosinase. Color scheme is as in Figure 3.

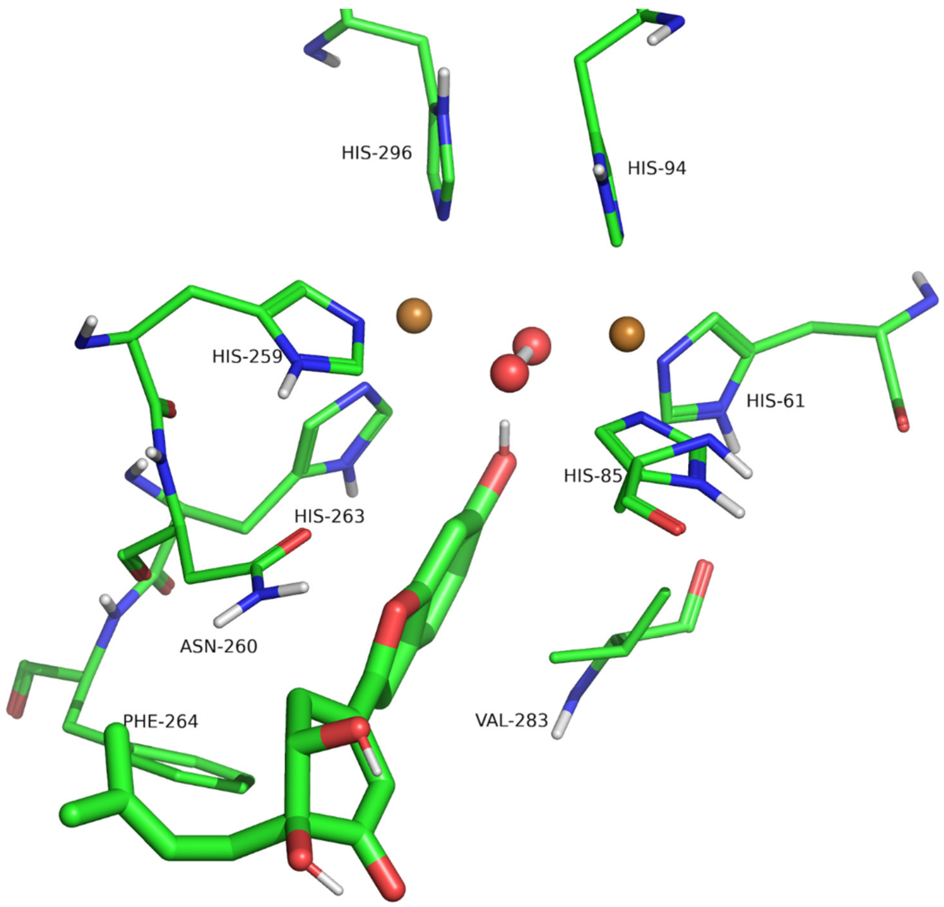

Figure S30. Docking of Streblus D to oxy-tyrosinase. Color scheme is as in Figure 3.

S3. Tables.

S3.1. Table S1.

**Table S1.** PubChem CID numbers correspond to the structure used to build the corresponding ligands.	
**PubChem CID**	**Compound name**
243	Benzoate
444539	Cinnamate
155565745	[2-(3-Methoxyphenoxy)-2-oxoethyl] 2,4-dihydroxybenzoate
155523436	2-(3-methoxyphenoxy)-2-oxoethyl-(E)-3-(4-hydroxyphenyl) acrylate
1555802	(Z)-2-(4-Hydroxybenzylidene)benzo[4,5]imidazo[2,1-b]thiazol-3(2H)-one
1555802	(Z)-2-(3,4-Dihydroxybenzylidene)benzo[4,5]imidazo[2,1-b]thiazol-3(2H)-one
1555802	(Z)-2-(2,4-Dihydroxybenzylidene)benzo[4,5]imidazo[2,1-b]thiazol-3(2H)-one
5396150	1,3-Dihydroxy-6H-benzo[c]chromen-6-one
5396150	1,3-Dihydroxy-8-methoxy-6H-benzo[c]chromen-6-one
76229	2’-(Hydroxymethyl)-[1,1’-biphenyl]-2,4-diol
2733300	trans-3,4-Diflurocinnamate
17842350	6-Fluoro-1H-indazole
24729247	7-Fluoro-1H-indazole
393037	4-Chloro-1H-indazole
17842471	6-Bromo-1H-indazole
20323899	7-Bromo-1H-indazole
227	2-Aminobenzoate
978	4-Aminobenzoate
5280445	Luteolin
4947	Propyl gallate
44170757	(E)-2-Acetyl-5-methoxyphenyl-3-(4-hydroxyphenyl)acrylate

44170757	(E)-2-Acetyl-5-methoxyphenyl-3-(4-methoxyphenyl)acrylate
44170757	E)-2-Isopropyl-5-methylphenyl-3-(4-hydroxyphenyl)acrylate
442458	Sanggenone C
528171	Oxyresveratrol
72276	L-Epicatechin
9064	Catechin
9994897	N-trans-Caffeoyltyramine
5280537	N-trans-feruloyltyramine
5372945	N-trans-Coumaroyltyramine
24801902	p-Aminophenyl-β-D-thiogalactopyranoside
638088	Streblus C
638088	Streblus D

S3.2. Table S2.

**Table S2. Variation of i_D to different $[I]_0$ and $[D]_0 = K_m^D$**								
**Competitive**		**Non competitive**		**Uncompetitive**		**Mixed**		
$[\mathbf{I}]_0(\mu\mathbf{M}$	$i_D(\%)$	$[I]_0(\mu M)$	$i_D(\%)$	$[I]_0(\mu M$	$i_D(\%)$	$[I]_0(\mu M$	$i_D^{M_1}(\%)$	$i_D^{M_2}(\%)$
0	0.00	0	0.00	0	0.00	0	0.00	0.00
10	14.21	10	27.10	10	15.84	10	19.82	38.66
15	19.92	15	35.79	15	22.00	15	27.05	48.59
30	33.25	30	52.71	30	36.03	30	42.61	65.37
45	42.80	45	62.57	45	45.76	45	52.70	73.89
60	49.96	60	69.03	60	52.92	60	59.78	79.04
72.50	54.70	72.50	72.92	72.50	57.58	72.50	64.24	82.01
100	62.50	100	78.79	100	65.16	100	71.26	86.27
200	76.95	200	88.14	200	78.87	200	83.22	92.63
300	83.37	300	91.77	300	84.84	300	88.16	94.96
-	-	-	-	-	-	500	92.55	96.91
-	-	-	-	-	-	700	94.56	97.78
Conditions: $[\mathbf{E}]_0 = \mathbf{10\ nM}$; $[\mathbf{D}]_0 = \mathbf{0.5\ mM}$								

S3.3. Table S3.

**Table S3.** i_D **values for** $[\mathbf{D}]_0 = \mathbf{2K_m^D}$ **and** $[\mathbf{D}]_0 = \mathbf{K_m^D}$		
**Type**	$[D]_0$	$i_D(\%)$
**Competitive**	$[D]_0 = K_m^D$	49.96
	$[D]_0 = 2K_m^D$	40.40
**Non-competitive**	$[D]_0 = K_m^D$	69.03
	$[D]_0 = 2K_m^D$	69.45
**Uncompetitive**	$[D]_0 = K_m^D$	52.92
	$[D]_0 = 2K_m^D$	60.34
**Mixed type (1)**	$[D]_0 = K_m^D$	59.78
	$[D]_0 = 2K_m^D$	55.88
**Mixed type (2)**	$[D]_0 = K_m^D$	79.04
	$[D]_0 = 2K_m^D$	81.39
Conditions: $[\mathbf{E}]_0 = \mathbf{10\ nM}$; $[\mathbf{D}]_0 = \mathbf{0.5\ mM}$ and $[\mathbf{I}]_0 = \mathbf{60\ \mu M}$		

S3.4. Table S4.

Table S4. Ligand-protein interactions observed in the docking conformations of the oxy and met forms of tyrosinase in the cases of competitive inhibition. Analysis of the interactions was carried out using PLIP software [14].

		OXY				MET				
**Figures**	**Compound name**	**Hydrogen bonds**	**Hydrophobic Interactions**	**π-π stacking**	**Others**	**Hydrogen bonds**	**Hydrophobic Interactions**	**π-π stacking**	**Salt Bridges**	**Metal Complexes**
**S2**	(Z)-2-(4-Hydroxybenzylidene)benzo[4,5]imidazo[2,1-b]thiazol-3(2H)-one		V248, N260, H263, F264, V283	H263			V248, N260, H263, F264, V283	H263 π-cation: H244		
**S3A,B**	(Z)-2-(3,4-Dihydroxybenzylidene)benzo[4,5]imidazo[2,1-b]thiazol-3(2H)-one	M280	V248, N260, H263, F264, V283	H263			V248, N260, H263, F264, V283	H263		Cu

**S4**	(Z)-2-(2,4-Dihydroxybenzylidene)benzo[4,5]imidazo[2,1-b]thiazol-3(2H)-one	H260	V248, N260, H263, F264, V283	H263						
**S6**	1,3-Dihydroxy-6H-benzo[c]chromen-6-one	H260	V248, H263, F264		Salt bridges: H85, H244	H260	V248, H263, F264, V283	H263	H85, H244	Cu
**S7**	1,3-Dihydroxy-8-methoxy-6H-benzo[c]chromen-6-one	H260	V248, H263, F264		Salt bridges: H85, H244	H260	V248, H263, F264, V283	H263	H85, H244	Cu
**S8**	2’-(Hydroxymethyl)-[1,1’-biphenyl]-2,4-diol		H85, N260	F264		H244, E256	V248, N260, V283	H244		

**S9**	trans-3,4-Diflurocinnamate		N260, H263, F264, V283	H263	Halogen bond: H279		F264, V283		H61, H85, H94, H259, H263, H296	Cu
**S17**	Sanggenone C	N81, H85, N260, V283, A323	A80, N81, V248, F264, V283, P284			N81, H85, V283, A323	A80, N81, V248, N260, F264, V283, P284, T324			Cu
**S19A,B**	L-Epicatechin	H244,E322	H85, V248			N81, C83, H85, H244	H85, V248			
**S20A,B**	Catechin	H244,E322	H85			N260	V248, N260, F264, V283, A286	H263 π-cation: H244		Cu

**S22A,B**	N-trans-Caffeoyltyramine		V248, N260, H263, F264, V283			T261	V248,N260, H263, F264, V283, A286		H263, F264	
**S23**	N-trans-feruloyltyramine	N81,H85, E322	H85, H263, F264, V283, A286	H263		N81, H85, N260	H85,N260, H263, F264, V283, A286	H263		
**S24**	N-trans-Coumaroyltyramine	H244, E322	H85, N260, H263, F264, V283, A286	H263		G281, V283	H263, F264, L275, P277, V283, A286	H263		Cu
**S29**	Streblus C	N260, M280	V248, F264, V283, A286	H263		H244, N260	V248, F264, V283			Cu
**S30**	Streblus D	N81, C83, H85, H244	H85, F264, V283			H244, E322	H85, V283	H263		Cu

S4. References

1. Rodríguez-López, J.N.; Ros, J.R.; Varón, R.; García-Cánovas, F. Oxygen Michaelis constants for tyrosinase. *Biochem. J.* **1993**, *293*, 859–866, doi:10.1042/bj2930859.

2. Fenoll, L.G.; Rodríguez-López, J.N.; García-Molina, F.; García-Cánovas, F.; Tudela, J. Michaelis constants of mushroom tyrosinase with respect to oxygen in the presence of monophenols and diphenols. *Int. J. Biochem. Cell Biol.* **2002**, *34*, 332–336, doi:https://doi.org/10.1016/S1357-2725(01)00133-9.

3. Jung, H.J.; Choi, D.C.; Noh, S.G.; Choi, H.; Choi, I.; Ryu, I.Y.; Chung, H.Y.; Moon, H.R. New Benzimidazothiazolone Derivatives as Tyrosinase Inhibitors with Potential Anti-Melanogenesis and Reactive Oxygen Species Scavenging Activities. *Antioxidants* 2021, *10*.

4. Lee, S.; Choi, H.; Park, Y.; Jung, H.J.; Ullah, S.; Choi, I.; Kang, D.; Park, C.; Ryu, I.Y.; Jeong, Y.; et al. Urolithin and Reduced Urolithin Derivatives as Potent Inhibitors of Tyrosinase and Melanogenesis: Importance of the 4-Substituted Resorcinol Moiety. *Int. J. Mol. Sci.* 2021, *22*.

5. Chen, J.; Ran, M.; Wang, M.; Liu, X.; Liu, S.; Yu, Y. Structure–activity relationships of antityrosinase and antioxidant activities of cinnamic acid and its derivatives. *Biosci. Biotechnol. Biochem.* **2021**, *85*, 1697–1705, doi:10.1093/bbb/zbab084.

6. Öztürk, C.; Bayrak, S.; Demir, Y.; Aksoy, M.; Alım, Z.; Özdemir, H.; İrfan Küfrevioglu, Ö. Some indazoles as alternative inhibitors for potato polyphenol oxidase. *Biotechnol. Appl. Biochem.* **2021**, *n/a*, doi:https://doi.org/10.1002/bab.2283.

7. Gheibi, N.; Taherkhani, N.; Ahmadi, A.; Haghbeen, K.; Ilghari, D. Characterization of inhibitory effects of the potential therapeutic inhibitors, benzoic acid and pyridine derivatives, on the monophenolase and diphenolase activities of tyrosinase. *Iran. J. Basic Med. Sci.* **2015**, *18*, 122–129.

8. Hashim, F.J.; Vichitphan, S.; Han, J.; Vichitphan, K. Alternative Approach

for Specific Tyrosinase Inhibitor Screening: Uncompetitive Inhibition of Tyrosinase by Moringa oleifera. *Mol.* 2021, *26*.

9. Lin, Y.-F.; Hu, Y.-H.; Lin, H.-T.; Liu, X.; Chen, Y.-H.; Zhang, S.; Chen, Q.-X. Inhibitory Effects of Propyl Gallate on Tyrosinase and Its Application in Controlling Pericarp Browning of Harvested Longan Fruits. *J. Agric. Food Chem.* **2013**, *61*, 2889–2895, doi:10.1021/jf305481h.

10. Yin, Z.-H.; Li, Y.-F.; Gan, H.-X.; Feng, N.; Han, Y.-P.; Li, L.-M. Synergistic effects and antityrosinase mechanism of four plant polyphenols from Morus and Hulless Barley. *Food Chem.* **2022**, *374*, 131716, doi:https://doi.org/10.1016/j.foodchem.2021.131716.

11. Kim, J.K.; Heo, H.-Y.; Park, S.; Kim, H.; Oh, J.J.; Sohn, E.-H.; Jung, S.-H.; Lee, K. Characterization of Phenethyl Cinnamamide Compounds from Hemp Seed and Determination of Their Melanogenesis Inhibitory Activity. *ACS Omega* **2021**, *6*, 31945–31954, doi:10.1021/acsomega.1c04727.

12. Sheng, Z.; Ge, S.; Xu, X.; Zhang, Y.; Wu, P.; Zhang, K.; Xu, X.; Li, C.; Zhao, D.; Tang, X. Design, synthesis and evaluation of cinnamic acid ester derivatives as mushroom tyrosinase inhibitors. *Medchemcomm* **2018**, *9*, 853–861, doi:10.1039/c8md00099a.

13. Nguyen, N.T.; Dang, P.H.; Nguyen, H.X.; Do, T.N. V; Le, T.H.; Le, T.Q.H.; Nguyen, M.T.T. Tyrosinase Inhibitors from the Stems of Streblus Ilicifolius. *Evid. Based. Complement. Alternat. Med.* **2021**, *2021*, 5561176, doi:10.1155/2021/5561176.

14. Adasme, M.F.; Linnemann, K.L.; Bolz, S.N.; Kaiser, F.; Salentin, S.; Haupt, V.J.; Schroeder, M. PLIP 2021: expanding the scope of the protein–ligand interaction profiler to DNA and RNA. *Nucleic Acids Res.* **2021**, *49*, W530–W534, doi:10.1093/nar/gkab294.
